# Gapless tunable intense terahertz pulse generation in strained diamond

**DOI:** 10.1038/s41377-025-02092-6

**Published:** 2026-03-31

**Authors:** Yudan Su, Yuxuan Wei, Chaonan Lin, Li Huang, Jiaming Le, Chong-xin Shan, A. H. Kung, Chuanshan Tian, Y. Ron Shen

**Affiliations:** 1 Department of Physics, State Key Laboratory of Surface Physics and Key Laboratory of Micro- and Nano-Photonic Structure (MOE), Fudan University, Shanghai, 200433 China; 2 Department of Physics, University of California, Berkeley, CA 94720 USA; 3 Zhangjiang Laboratory, Shanghai, 201204 China; 4 Henan Key Laboratory of Diamond Optoelectronic Materials and Devices, School of Physics and Microelectronics, Zhengzhou University, Zhengzhou, 450052 China; 5 Institute of Quantum Materials and Physics, Henan Academy of Sciences, Zhengzhou, 450046 China

**Keywords:** Terahertz optics, Nonlinear optics

## Abstract

We demonstrate collinear phase matching of transient coherent Stokes Raman scattering (CSRS) in strained diamond, enabling generation of intense, high-quality, femtosecond THz pulses over a gapless frequency band from 5 to beyond 15 THz. Our experimental results confirm this mechanism, supported by the theoretical calculations that agrees well with measurements. The theoretical model also provides a clear picture on how the input pulses should be adjusted to optimize the THz output from a diamond of given length.

## Introduction

The generation of high-intensity, ultrashort, coherent terahertz pulses in the 5-15 THz frequency range presents a significant challenge in the development of strong field terahertz sources^1–7^. This frequency range, coinciding with the reststrahlen band of most nonlinear optical crystals, is commonly referred to as the ‘new THz gap’^7^. Such THz sources are critical for selective excitation of matter and optical manipulation of structures and dynamics of quantum materials^1–4,8,9^ and biomolecules^10^. For instance, a transient superconducting state in K_3_C_60_ can be more easily induced using a 10 THz pulse than a mid-infrared (MIR) pulse^4,11^, and resonant excitation of the 10.8 THz chiral phonon mode in rare-earth halides can create effective magnetic fields exceeding 1 tesla^1^. Several techniques have been developed to generate energetic THz pulses in this frequency range: difference frequency generation (DFG) in organic crystals^6,12,13^, asymmetric ionization in air plasmas^14,15^, and spin Hall effect in ferromagnetic films^16,17^. However, limitations exist with each approach. In organic crystals, there are only a few narrow transparent windows above 5 THz^14,18^, and instability and high cost of organic crystals hinder their use^19^. The THz source based on air plasmas or ferromagnetic films can generate ultra-broadband THz but suffers from low energy density at specific frequencies, making them less suitable for strong excitation of narrow material resonances.

Recently, we have introduced a novel scheme to generated intense broadband THz pulse based on Raman-resonance-enhanced four-wave mixing (R-FWM), or known as coherent Stokes Raman scattering (CSRS), in diamond^20^. The process starts with coherent Raman excitation of phonons in a medium by a pair of input light pulses at frequencies *ω*_1_ and *ω*_2_, followed by beating of the coherent phonons with another input pulse at *ω*_MIR_ to generate a THz pulse at *ω*_MIR_ - (*ω*_1_ -*ω*_2_). Through Raman resonance enhancement with sufficient input pulse intensities, the induced nonlinear polarization of CSRS can be comparable to that of DFG in second-order nonlinear crystals. The process has several important advantages. The THz generation from diamond is continuously tunable from 5 THz to MIR without any gap. The spectral and temporal characterization of the THz output pulses in ref. ^20^ give the evidence of stable and have high-quality spatial and temporal modes of CSRS THz source. With femtosecond (fs) input pulses at *ω*_MIR_, ultrashort THz pulses can be generated with a controllable carrier-envelope phase (CEP) offset, and a peak field strength over 1 MV cm^−1^ at focus, thus being able to bridge the ‘new THz gap’^20^. However, phase matching for CSRS in normal diamond is possible only with non-collinear geometry, which limits the interaction length and hence, the THz output energy. The noncollinear geometry also causes inconvenience in experimental arrangement as tuning of the THz frequency requires readjustment of the beam geometry, and the spatial chirp of the THz output pulse makes it more difficult to focus.

In this work, we show that collinear phase matching of CSRS can be achieved over 5 to >15 THz in a stressed diamond. We found experimentally that with collinear phase matching, the output pulse energy at 10 THz from a 2-mm thick diamond could be three times that of the noncollinear case (30 nJ versus 10 nJ, corresponding to a peak electric field strength of ~2.3 MV cm^−1^ versus ~1 MV cm^−1^ at focus with the focal area approaching the diffraction limit); the output was limited because the pump pulse intensity for Raman excitation was limited by optical damage in diamond and was strongly depleted in propagation through diamond. We have investigated theoretically and experimentally the pump depletion effect on THz generation. The theory predicts realistic input conditions for optimal THz generation in a diamond of given length and how the THz output increases with the diamond length.

## Theoretical background

**Fig. 1:**
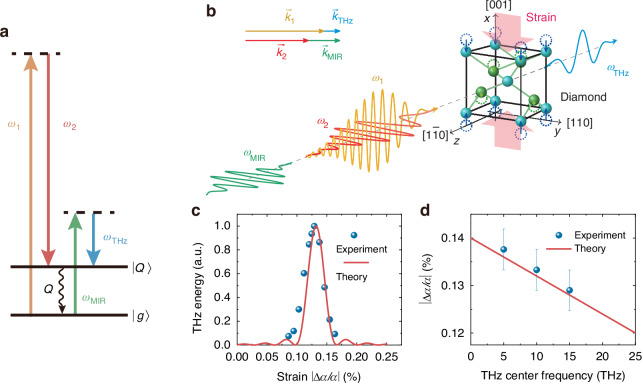
THz generation by CSRS in strained diamond. **a** Energy level diagram of CSRS. **b** Schematic of the experimental geometry used to study CSRS in diamond under a uniaxial stress. **c** Theoretical and experimental results of THz output energy versus strain at 10 THz from diamond under uniaxial stress. **d** Strain (|Δ*a/a* | ) required to achieve collinear phase matching of CSRS in diamond at different THz frequencies. The experimental data points refer to the center frequencies of the generated THz pulses

We briefly sketch the theory of CSRS in diamond below. As depicted in Fig. 1a, two input pulsed fields, ${\vec{E}}_{i}\left(\vec{r},t\right)={\vec{A}}_{i}\left(\vec{r},t\right){e}^{i{\vec{k}}_{i}\cdot \vec{r}-i{\omega }_{i}t}$, (*i* = 1, 2), coherently and resonantly excite the Raman-active phonon of diamond, $\overleftrightarrow{{\rm{{\mathbb{Q}}}}}\left(\vec{r},t\right)=\overleftrightarrow{Q}\left(\vec{r},t\right){e}^{i{\vec{k}}_{Q}\vec{r}-i{\omega }_{Q}t}$, with ${\vec{k}}_{Q}={\vec{k}}_{1}-{\vec{k}}_{2}$ and ${{\omega }_{Q}=\omega }_{1}-{\omega }_{2}$ at 40 THz (1332 cm^−1^). A third input pulse, ${\vec{E}}_{\rm{MIR}}\left(\vec{r},t\right)={\vec{A}}_{\rm{MIR}}\left(\vec{r},t\right){e}^{i{\vec{k}}_{\rm{MIR}}\cdot \vec{r}-i{\omega }_{\rm{MIR}}t}$, then beats with $\overleftrightarrow{{\rm{{\mathbb{Q}}}}}$ and generates a THz output pulse with the center frequency at ${\omega }_{\rm{THz}}={\omega }_{\rm{MIR}}-\left({\omega }_{1}-{\omega }_{2}\right)$. We consider the case where ${\vec{E}}_{1}$ and ${\vec{E}}_{2}$ are picosecond pulses with pulse widths shorter than phonon dephasing time of diamond so that the excitation of $\overleftrightarrow{{\rm{{\mathbb{Q}}}}}({\rm{t}})$ is transient; ${\vec{E}}_{\rm{MIR}}$ is a femtosecond pulse much shorter than $\overleftrightarrow{{\rm{{\mathbb{Q}}}}}({\rm{t}})$ and its beating with $\overleftrightarrow{{\rm{{\mathbb{Q}}}}}({\rm{t}})$ to generate ${\vec{E}}_{\rm{THz}}$ could be considered a mapping process with ${\vec{A}}_{\rm{THz}}\left(\vec{r},t\right){\propto }{\vec{A}}_{\rm{MIR}}\left(\vec{r},t\right)$ and ${\omega }_{\rm{MIR}}$ down-converted to ${\omega }_{\rm{THz}}$.

With a particular set of polarized input pulses as described in Fig. 1b, the coupled equations describing a collinear transient CSRS process along *z* axis are^21,22^

1a $${\partial }_{t}Q+\gamma Q=\frac{i{\epsilon }_{0}R}{2{\omega }_{Q}}{A}_{1}{A}_{2}^{* }$$

1b $${\partial }_{z}{A}_{1}+\frac{1}{{v}_{g1}}{\partial }_{t}{A}_{1}=i\frac{{\omega }_{1}R}{2{n}_{1}c}{A}_{2}Q$$

1c $${\partial }_{z}{A}_{2}+\frac{1}{{v}_{g2}}{\partial }_{t}{A}_{2}=i\frac{{\omega }_{2}R}{2{n}_{2}c}{A}_{1}{Q}^{* }$$

1d $${\partial }_{z}{A}_{{\rm{THz}}}+\frac{1}{{v}_{g,{\rm{THz}}}}{\partial }_{t}{A}_{\rm{THz}}=i\frac{{\omega }_{{\rm{THz}}}R}{2{n}_{{\rm{THz}}}c}{Q}^{* }{A}_{{\rm{MIR}}}{e}^{i\Delta {kz}}$$

Here, slowly varying amplitude approximation is used. *R* and γ are the Raman matrix element and the phonon damping rate of diamond, respectively, $\Delta k={k}_{\rm{MIR}}-{k}_{\rm{THz}}-{k}_{Q}={k}_{\rm{MIR}}-{k}_{\rm{THz}}-\left({k}_{1}-{k}_{2}\right)$ is the phase mismatch, and *n*_*i*_ and ${v}_{{gi}}=\frac{c}{{n}_{{gi}}}$ are the refractive index and group velocity of the *ω*_*i*_ wave, respectively. The values of these parameters are provided in Sec. 1 of Supplementary Information (SI). Note that, in Eq. (1d), when the THz carrier frequency ${\omega }_{{\rm{THz}}}$ is smaller than the bandwidth of the fs THz pulses, the slowly varying amplitude approximation in time breaks down. The corrected Eq. (1d) is provided in SI Sec. 2. The three input pulses on diamond, specified by *i* = 1, 2, and MIR, are described by ${A}_{i}\left(\vec{r},t\right)={A}_{i}\left(z=0,\vec{{\rho }},t\right)={A}_{{im}}\left(\vec{{\rho }}\right){f}_{i}\left(t-\Delta {t}_{1i}\right)$ with ${A}_{{im}}\left(\vec{{\rho }}\right)$ denoting the spatial profile and ${f}_{i}\left(t-\Delta {t}_{1i}\right)$ the temporal profile, where $\Delta {t}_{1i}$ is the time lag of the peak of the *i*-pulse from the peak of the *ω*_1_ pulse and $\Delta {t}_{11}=0$. In the simple case where the changes of inputs and differences of group velocities of pulses are negligible, the *ω*_*i*_ fields in the crystal can be written by ${A}_{i}\left(z,\vec{{\rho }},t\right)={A}_{{im}}\left(\vec{{\rho }}\right){f}_{i}\left(t-\Delta {t}_{1i}-z/{v}_{g}\right)$, and from Eq. (1a), we find:

2 $$Q\left(z,\vec{{{\rho }}},t\right)=\frac{i{\epsilon }_{0}R}{2{\omega }_{Q}\gamma }{f}_{Q}\left(t-\frac{z}{{v}_{g}}\right){A}_{1m}\left(\vec{{{\rho }}}\right){A}_{2m}^{* }\left(\vec{{{\rho }}}\right)$$

and from Eq. (1d) with phase matching ($\varDelta k=0$), the THz output field amplitude at *z* = *L* is:

3 $${A}_{\rm{THz}}\left(z=L,\vec{{{\rho }}},t\right)=\frac{{\omega }_{{\rm{THz}}}{\chi }_{{st}}^{\left(3\right)}}{2{n}_{{\rm{THz}}}c}{f}_{Q}\left(\Delta {t}_{\rm{MIR},0}\right){A}_{1m}\left(\vec{{{\rho }}}\right){A}_{2m}^{* }\left(\vec{{{\rho }}}\right){A}_{{\rm{MIR}}}\left(\vec{{{\rho }}}\right){f}_{\rm{MIR}}\left(t-\frac{L}{{v}_{g}}\right)L$$

with ${\chi }_{{st}}^{\left(3\right)}=\frac{{R}^{2}}{2{\varepsilon }_{0}\gamma {\omega }_{Q}}$. The corresponding THz output energy per pulse is given by^20^

4 $$W\left({\omega }_{\mathrm{THz}}\right)\approx \left(1-{R}_{\mathrm{THz}}\right)\frac{{\omega}_{\rm{THz}}^{2}}{16{\epsilon}_{0}^{2}{c}^{4}{n}_{1}{n}_{2}{n}_{\rm{MIR}}{n}_{\rm{THz}}}{\left|{f\left(\Delta {t}_{{\rm{MIR}},0}\right)\chi}_{st}^{\left(3\right)}\right|}^{2}\frac{W\left({\omega }_{1}\right)W\left({\omega }_{2}\right)W\left({\omega}_{\rm{MIR}}\right)}{{T}^{2}{S}^{2}}{L}^{2}$$

where *W* denotes the pulse energy, *T* and *S* are the effective time duration and the beam area of the *ω*_1_ and *ω*_2_ input pulses, respectively, and $(1-{R}_{{\rm{THz}}})$ is the transmission coefficient for THz emission from diamond.

In our study here, however, the situation is more complicated. First, the input intensities are high, so that for diamond length *L* over ~2 mm, pump depletion of the *ω*_1_ pulse and gain of the *ω*_2_ pulse cannot be ignored, and then, the group velocity mismatch (GVM) between pulses are also not negligible. Moreover, we consider chirp-stretched *ω*_1_ and *ω*_2_ input pulses with the same chirp rate for resonant Raman excitation, but, with strong pump depletion and GVM, the time lag between two pulses $\Delta {t}_{12}$, controlling the deviation of Raman excitation from resonance, also become an important parameter for THz output optimization. To solve Eq. (1) with these considerations, we have to resort to numerical calculation, the result of which will be discussed in a later section.

## Experimental arrangement

Details of our experimental arrangement were given in Sec. 3 of SI. Briefly, a 33-fs, 5-W, 1-kHz Ti:sapphire laser system was used to pump two optical parametric amplifiers (OPAs) seeded by a common white light source. The pair of 50-fs signal (*ω*_1_) and idler (*ω*_2_) pulses at 6900 and 5600 cm^−1^, respectively, derived from OPA1 and chirp-stretched to ~1 ps by passing them through dispersive ZnSe rods, were used to resonantly excite the 40 THz phonon mode of diamond (Temporal profiles and the chirps of the two pulses are described in Sec. 4 of SI). The chirped frequency of *ω*_*i*_ pulse at time *t* was ${\omega }_{i}+k\left(t-\Delta {t}_{1i}\right)$ with $k=7\,{\rm{THz}}/{\rm{ps}}\times 2{\rm{\pi }}$. With *ω*_1_-*ω*_2_ set at the phonon frequency of diamond, the frequency detuning of Raman excitation was $k\Delta {t}_{12}$. Thus, adjusting $\varDelta {t}_{12}$ allows control of detuning Raman excitation from resonance. The MIR (${\omega }_{\rm{MIR}}$) pulses with 50 fs pulse width were generated from a DFG stage following OPA2 with its center frequency tunable from 45 THz to 65 THz; they were collinearly combined with the *ω*_1_ and *ω*_2_ pulses after a controllable time delay $(\Delta {t}_{1,{\rm{MIR}}})$. (See Fig. 1(b).) The beam areas of *ω*_1_, *ω*_2_, and MIR pulse were *π*(185 × 165) μm^2^/2, *π*(235 × 215) μm^2^/2 and *π*(140 × 120) μm^2^/2, respectively.

The sample used in our experiment was a 2×2×2 mm^3^ CVD diamond cube; It was sandwiched between two steel plates through which compressive stress was applied on the diamond along its [001] axis. In strain-free diamond, the phase mismatch vector of collinear CSRS process is $\Delta k={k}_{1}+{k}_{{\rm{T}}{\rm{H}}{\rm{z}}}-{k}_{2}-{k}_{{\rm{M}}{\rm{I}}{\rm{R}}} > 0$. By applying uniaxial strain to diamond along [001] direction (*x*-axis in Fig. 1 (b)), the refractive index *n* of *x*-polarized light in diamond will decrease corresponding to shorter wave vectors of light with polarization along *x*-axis, namely *k*_1_ and *k*_THz_. Thus, by apply appropriate strain to diamond, Δ*k* = 0 in collinear geometry can be achieved. Characterization of the THz output was carried out following the same procedures described in ref. ^20^.

## Results

Figure 1(c) shows the variation of the measured output energy per pulse at 10 THz in response to the exerted strain on the diamond cube. The data match well with the predicted collinear phase matching curve. With the strain varied from 0.14% to 0.12%, the phase-matched THz frequency is predicted to shift from 5 to 25 THz as plotted in Fig. (1d). Measured strains for phase-matched THz generation at 5, 10, and 12 THz are close to the predicted values. The theoretical calculation about strain optimization and the effect of strain inhomogeneity are shown in Sec. 5 of SI.

**Fig. 2:**
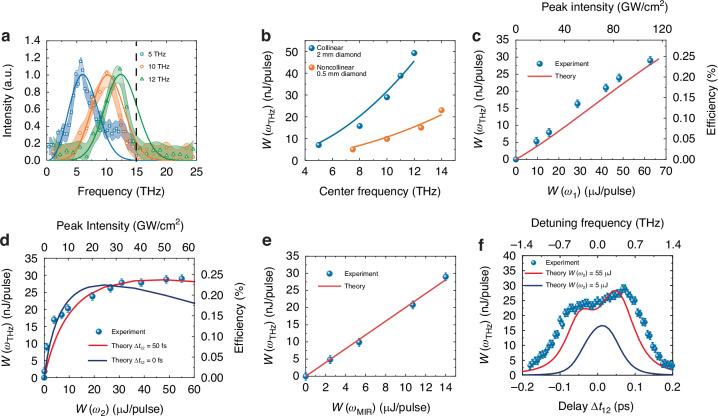
Experimental results on THz generation in strained diamond. **a** Measured output spectra of CSRS at three different center THz frequencies. The solid curves are the THz spectra expected from the down-conversion of the MIR spectra. The dashed line indicates the absorption cutoff edge of the PE filter. **b** Measured THz output pulse energy (dots) versus THz center frequency in collinear and noncollinear (ref. ^20^) phase-matching beam geometries. Solid curves depict the fits of the expected ${\omega }_{\rm{THz}}^{2}$ dependence. **c**–**e** Dependences of 10-THz output energy from a 2-mm diamond and corresponding peak intensities (upper axes) on input pulse energies *W*(*ω*_1_), *W*(*ω*_2_*)*, and *W*(*ω*_MIR_), respectively. **f** The 10-THz output pulse energy versus $\varDelta {t}_{12}$. The theoretical curves in **c**–**f** are from numerical solution of Eq. (1a–d)

Tuning of collinear phase-matched THz output frequency was experimentally achieved by simultaneously tuning the input MIR frequency and adjusting the strain on the diamond. The observed THz spectra in the 5 to 12 THz range are shown in Fig. 2a. They are supposed to be down-converted copies of the input MIR pulse spectra (The 60 fs MIR pulse is mapped to a 60 fs THz pulse). Unfortunately, in our experiment adopting the same detection scheme as in the previous CSRS measurement with noncollinear phase matching geometry^20^, the quality of the measured spectra was significantly deteriorated by the ineffectiveness of the polyethylene (PE) filter used to block the MIR input that propagated collinearly with the THz output. As seen in Fig. 1c, it is more troublesome for the 5 THz pulse spectrum at the high-frequency range end because of its relatively weak signal there. Also, the absorption cutoff of PE above 15 THz^23^ prevented us from carrying out measurements of THz output at higher frequencies.

In our attempt to maximize the THz output from the 2-mm long diamond, we used pulse energies of *W*(*ω*_1_) = 64 μJ, *W*(*ω*_2_) = 55 μJ, and *W*(*ω*_MIR_) = 14 μJ for the three input pulses. (With the spatial and temporal profiles of the pulses mentioned earlier, the peak intensities of the pulses on the diamond were *I*_*m*_(*ω*_1_) = 110 GW cm^−2^, *I*_*m*_(*ω*_2_) = 55 GW cm^−2^, *I*_*m*_(*ω*_MIR_) = 0.7 TW cm^−2^) The first two were limited by optical damage of diamond and the other one was limited by available pulse energy from our setup. The optimum time lag for the MIR pulse was $\varDelta {t}_{1,{\rm{MIR}}}=0.6\,{\rm{ps}}$, see SI Sec 6. We found however that the THz output could be further enhanced by 10% if $\varDelta {t}_{12}$ was adjusted to 50 fs equivalent to detuning of Raman resonant excitation by 0.35 THz. As we shall explain in the next section, this was because the weaker Raman excitation created a more extended phonon distribution in the diamond that, in beating with the MIR pulse, generated more THz output.

Under the setting mentioned above, we have measured the THz output pulse energy versus the center THz frequency from our 2-mm properly strained diamond. The result is displayed in Fig. 2b. At 10 THz, the output reaches 30 nJ per pulse, which corresponds to a MIR-to-THz energy conversion efficiency of 0.25% (photon conversion efficiency of 1.25%). Extrapolation to higher THz frequencies suggests that the energy conversion efficiency can reach ~0.56% at 15 THz, which is much higher than the near-IR to THz energy conversion efficiency of DFG from GaSe (0.07%, scaled from THz generation at higher frequencies in ref. ^24^). In SI sec. 13 we summarize different THz pulse generation schemes suitable for small laboratories for comparison. The transverse spatial profile of the THz output was found to be near-Gaussian in two orthogonal directions and could be focused by a 2:1 parabolic mirror telescope to a near-diffraction-limited spot of π(55 × 75)/2 μm^2^ (1/e^2^ radius), see SI sec. 7. Thus, the focused 30-nJ, 60-fs 10-THz pulse could have a peak electric field of 2.3 MV cm^−1^.

As a comparison, we plot in Fig. 2b the THz output pulse energy versus frequency measured from CSRS in a 0.5-mm diamond using the noncollinear phase matching geometry with the same set of pump sources^20^. It appears only ~3 times less than the collinearly phase-matched case in a 2-mm diamond, while under the pump depletion-less limit, the former should be less than (0.5/2)^2^ = 1/16 of the latter. Clearly, the pump depletion-less assumption is not valid in the latter case.

We have also measured how THz output energy per pulse, *W*(*ω*_THz_), depends on the individual variation of input powers *W*(*ω*_1_), *W*(*ω*_2_), and *W*(*ω*_MIR_) in the collinear phase-matching case. The result is depicted in Fig. 2(c)-(e). While *W*(*ω*_THz_) appears linearly proportional to *W*(*ω*_1_) and *W*(*ω*_MIR_), a saturation-like behavior is seen in the plot of *W*(*ω*_2_). (Fig. 2d). Upon varying $\varDelta {t}_{12}$, we observed a broad asymmetrically split peak around $\varDelta {t}_{12}=0$ with optimal values at +50 fs, shown in Fig. 2(f). We shall defer the explanation of these results to the next section.

**Fig. 3:**
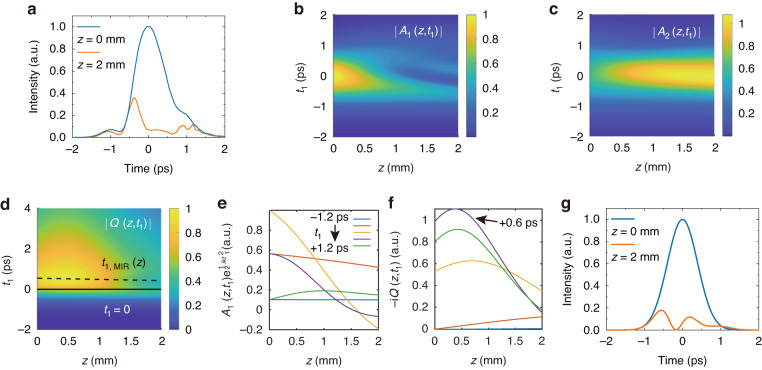
Evolution of optical and phonon waves in CSRS process. **a** Measured average |*A*_1_(*z,t*_1_)|^2^ around the center of the beam overlapping area as a function of time at the entrance (*z* = 0 mm) and the exit (*z* = 2 mm) of a 2 mm diamond. **b**–**d** Calculated evolution of |*A*_1_(*z,t*_1_)|, |*A*_2_(*z,t*_1_)| and |*Q*(*z*,*t*_1_)| in z and ${t}_{1}$ with ${t}_{1}=t-\frac{z}{{v}_{g1}}$. The trajectory of the fs MIR pulse ${t}_{{\rm{MIR}}}(z)$ is marked by the dash line at ${t}_{1}={t}_{\rm{MIR}}\left(z\right)-\frac{z}{{v}_{g1}}\approx \Delta {t}_{1,{\rm{MIR}}}$ in **d**. It nearly goes through the maxima of |*Q*(*z, t*_1_)| of all *z* to maximize the THz output. **e**, **f** Calculated evolutions of $A\left(z,{t}_{1}\right){e}^{\frac{1}{2}{ik}{t}_{1}^{2}}$ and -*iQ*(*z*,*t*_1_) for *t*_1_ = -1.2 ps, -0.6 ps, 0 ps, +0.6 ps and +1.2 ps. **g** Calculated temporal profiles of |*A*_1_(*z,t*_1_)|^2^ at *z* = 0 mm and 2 mm obtained from line cuts of 3D plot in (b)

Strong energy depletion of the *ω*_1_ pulse resulting from CSRS in passing through the 2-mm diamond sample was experimentally observed. The depletion of *W*(*ω*_1_), measured directly by an IR power meter, reached 56% when *W*(*ω*_1_) and *W*(*ω*_2_) were at their highest values displayed in Fig. 2(d) and (e). At the beam center, the depletion of *W*(*ω*_1_) was more significant. Figure 3(a) presents the observed temporal profile of the intensity, |*A*_1_(*z,t*_1_)|^2^, averaged over an area of ~20 μm in a radius around the beam center, before (*z* = 0 mm) and after (*z* = 2 mm) the *ω*_1_ pulse passing through the diamond, measured by the intensity cross-correlation method (see SI section 4). Overall depletion at the beam center, calculated from integration of |*A*_1_(*z,t*_1_)|^2^ over *t*_1_, exceeds 78%. The pulse is nearly fully consumed except for a small portion at the pulse front.

## Discussion

For a quantitative understanding of the experimental results, we have numerically solved the coupled equations in Eq. (1). Alongside the numerical results in this section, we shall describe physically how the results can be anticipated. We shall also discuss for a given length of diamond crystals how the THz output can be maximized by tuning the input parameters, what the appropriate diamond length can be used in practice, and how one can further increase the THz output with CSRS in diamond.

### Numerical Calculation of Optical and Phonon Waves Evolution

In our numerical calculation, we approximated the chirped input pulses at *ω*_1_ and *ω*_2_ as Gaussian-like: ${A}_{i}\left(\vec{\rho },z=0,t\right)={A}_{{im}}\left(\vec{\rho }\right){e}^{-\frac{{\left(t-\Delta {t}_{i1}\right)}^{2}}{{\tau }_{i}^{2}}}{e}^{-i\frac{1}{2}k{\left(t-\Delta {t}_{i1}\right)}^{2}}\left(i=\mathrm{1,2}\right)$ with ${A}_{{im}}\left(\vec{\rho }\right)={A}_{{im}0}{e}^{-\frac{{x}^{2}}{{w}_{x,i}^{2}}-\frac{{y}^{2}}{{w}_{y,i}^{2}}}$, τ_1_ = 0.8 ps, τ_2_ = 1 ps, $k=7.0{\rm{THz}}/{\rm{ps}}\times 2\pi$ (see SI Sec. 4), and ${w}_{{ix}},{w}_{{iy}}$ are provided in the Experiment arrangement section. The solution of Eq. (1a-c) for CSRS in a diamond of length *L* yielded the wave amplitudes $Q\left(z,\vec{\rho },t\right)$, ${A}_{1}\left(z,\vec{\rho },t\right)$, and ${A}_{2}\left(z,\vec{\rho },t\right)$. Knowing that $Q\left(z,\vec{\rho },t\right)$ hardly changed when the fs MIR pulse passed through the diamond and depletion of ${A}_{{\rm{MIR}}}\left(z,\vec{\rho },t\right)$ was negligible, we found from Eq. (1d),

5 $${A}_{{\rm{THz}}}\left(z=L,\vec{\rho },t\right)=i\frac{{\omega }_{\rm{THz}}R}{2{n}_{\rm{THz}}c}{A}_{{\rm{MIR}}}\left(z=L,\vec{\rho },t\right){\int }_{0}^{L}Q\left(z,\vec{\rho },{t}_{\rm{MIR}}(z)\right){dz}$$

where ${A}_{{\rm{MIR}}}\left(z,\vec{\rho },t\right)={A}_{{\rm{MIR}},0}\left(\vec{\rho }\right){{\rm{e}}}^{-\frac{{\left(t-{t}_{\rm{MIR}}(z)\right)}^{2}}{{\tau }_{\rm{MIR}}^{2}}}$ with ${t}_{\rm{MIR}}\left(z\right)=\Delta {t}_{1,{\rm{MIR}}}+\frac{z}{{v}_{{\rm{MIR}}}}$. The output THz pulse energy is:

6 $$W\left({\omega }_{\mathrm{THz}}\right)=(1-{R}_{\mathrm{THz}})\frac{{\epsilon }_{0}{\omega }_{\rm{THz}}^{2}{R}^{2}}{2{n}_{\rm{THz}}c}\iiint {\left|{{\rm{A}}}_{\mathrm{MIR}}\left({\rm{z}}={\rm{L}},\vec{{{\rho }}},{\rm{t}}\right){\int }_{0}^{{\rm{L}}}{\rm{Q}}\left({\rm{z}},\vec{{{\rho }}},{{\rm{t}}}_{\rm{MIR}}\left({\rm{z}}\right)\right)dz\right|}^{2}{dtd}\vec{\rho }$$

Because the THz generation is from the beating of *Q* with an essentially constant *A*_MIR_ along the length of the diamond, the evolution of *Q* in *t* and *z* is obviously the most important. To see more clearly how *Q*(*z*,*t*) evolves from transient coherent Raman excitation in diamond, we consider wave interaction along *z* in a small region of the beam overlapping area where the input beam intensity can be approximated as homogeneous so that the $\rho$ dependence can be ignored. In the numerical calculation to be presented, we took the region to be a circular area of 20 μm in radius at the center of the beam overlapping area with peak input beam intensities *I*_*m*_(*ω*_1_) = 115 GW cm^−2^ and *I*_*m*_(*ω*_2_) = 55 GW cm^−2^; other input parameters in the calculation were the same as those stated in the Experimental section. For better illustration, we transformed Eq. 1(a-c) into a frame propagating at *v*_*g*1_ with ${t}_{1}\equiv t-\frac{z}{{v}_{g1}}$:

7a $$\frac{\partial {iQ}\left(z,{t}_{1}\right)}{\partial {t}_{1}}+\gamma \left({iQ}\left(z,{t}_{1}\right)\right)=-\frac{{\epsilon }_{0}R}{2{\omega }_{Q}}{A}_{1}\left(z,{t}_{1}\right){A}_{2}^{* }\left(z,{t}_{1}\right)$$

7b $$\frac{\partial {A}_{1}\left(z,{t}_{1}\right)}{\partial z}=\frac{{\omega }_{1}R}{2{n}_{1}c}{A}_{2}\left(z,{t}_{1}\right){iQ}\left(z,{t}_{1}\right)$$

7c $$\frac{\partial {A}_{2}\left(z,{t}_{1}\right)}{\partial z}+\left(\frac{1}{{v}_{g2}}-\frac{1}{{v}_{g1}}\right){\partial }_{t}{A}_{2}=\frac{{\omega }_{{\rm{THz}}}R}{2{n}_{{\rm{THz}}}c}{A}_{1}\left(z,{t}_{1}\right){\left({iQ}\left(z,{t}_{1}\right)\right)}^{* }$$

In general, ${A}_{1}{e}^{\frac{1}{2}{ik}{t}_{1}^{2}},{A}_{2}{e}^{\frac{1}{2}{ik}{\left({t}_{1}-\Delta {t}_{12}\right)}^{2}}$, and -*iQ* are complex, but for z < 2 mm where the effect of ${v}_{g1}{\ne }{v}_{g2}$ is not yet appreciable, the phase factors of ${A}_{1}$ and ${A}_{2}^{* }$ arising from chirping nearly cancel each other in Eq. (7a) for $\varDelta {t}_{12}\lesssim 50\,{\rm{fs}}$, and then, ${A}_{1}{e}^{\frac{1}{2}{ik}{t}_{1}^{2}},{A}_{2}{e}^{\frac{1}{2}{ik}{\left({t}_{1}-\Delta {t}_{12}\right)}^{2}}$, and -*iQ* are predominantly positive real values. How $\varDelta {t}_{12}$ and ${v}_{g1}{\ne }{v}_{g2}$ could affect phases of the fields is described in SI section 8.

## Dynamics of Transient photon-phonon coupling

The results of our numerical calculation are presented via 3D plots for $|{A}_{1}\left(z,{t}_{1}\right)|,|{A}_{2}\left(z,{t}_{1}\right)|$ and |*Q*(*z,t*_1_)| in Fig. 3(b-d), respectively, which describe how they evolve in *t* and *z*. We discuss in the following how physically the results come about, with particular emphasis on the depletion of $|{A}_{1}\left(z,{t}_{1}\right)|$ and growth of |*Q*(*z,t*_1_)|.

1. At the leading edge of input pulses (*t*_1_ < 0.5 ps) when both $|{A}_{1}\left(z,{t}_{1}\right)|$ and $|{A}_{2}\left(z,{t}_{1}\right)|$ are weak, their changes through Raman excitation in diamond are not appreciable and |*Q*(*z,t*_1_)| is also not significant.
2. Close to the front end of the diamond (z ~ 0), depletion of $|{A}_{1}|$ and gain of $|{A}_{2}|$ are also hardly appreciable due to the short Raman interaction length. As the excitation pules pass through *z* = 0^+^, | *Q*(*z,t*_1_)| increases rapidly with time, in accordance with Eq. (7a). (See Fig. 3d.) Because the phonon relaxation time is much longer than the input pulse width, |*Q*(*z,t*_1_)| keeps on being excited until $|{A}_{1}\left(z,{t}_{1}\right)|$ and $|{A}_{2}\left(z,{t}_{1}\right)|$ pulses are essentially over (at ${t}_{1} \sim 0.6\,{\rm{ps}}$ for your pulse width) and then decays through relaxation.
3. For the main part of the pulses, depletion of $|{A}_{1}\left(z,{t}_{1}\right)|$ increases significantly with *z* according to Eq. (7b) and correspondingly, both ${A}_{2}\left(z,{t}_{1}\right)$ and -*iQ*(*z,t*_1_) grow. We have, from Eq. (7a), $-{iQ}\left(z,{t}_{1}\right)\propto {\int }_{-{\infty }}^{{t}_{1}}{A}_{1}{A}_{2}^{* }d{t}_{1}^{{\prime} }\propto {\int }_{-{\infty }}^{{t}_{1}}\sqrt{{N}_{1}\cdot {N}_{2}}d{t}_{1}^{{\prime} }$, ${\rm{where}}$ ${N}_{i}\left(z,{t}_{1}\right)\propto {|{A}_{i}\left(z,{t}_{1}\right)|}^{2}/\hslash {\omega }_{i}$ is the photon number associated with ${|{A}_{i}\left(z,{t}_{1}\right)|}^{2}$ at ${\omega }_{i}$. Because the total photon number should be conserved in the Raman process, that is, ${N}_{1}\left(z,{t}_{1}\right)+{N}_{2}\left(z,{t}_{1}\right)={N}_{1}\left(0,{t}_{1}\right)+{N}_{2}\left(0,{t}_{1}\right)$, and ${N}_{1}\left(0,{t}_{1}\right) > {N}_{2}\left(0,{t}_{1}\right)$ in our case, decrease of ${N}_{1}$ and corresponding increase of ${N}_{2}$ along *z* must lead to increase of $-{iQ}(z,{t}_{1})$, until it reaches its maximum when ${N}_{1}={N}_{2}=({N}_{1}+{N}_{2})/2$. This happens around z = 0.5 mm as seen in Fig. 3(d). With lower input of ${A}_{1}\left(z=0,{t}_{1}\right)$ and ${A}_{2}\left(z=0,{t}_{1}\right)$, the maximum shifts to larger *z* due to lower Raman excitation.
4. Traveling further down along *z*, $|{A}_{1}\left(z,{t}_{1}\right)|$ decays toward zero and $|{A}_{2}\left(z,{t}_{1}\right)|$ still keeps increasing. Because $-{iQ}(z,{t}_{1})$ is proportional to the time integration of ${A}_{1}{A}_{2}^{* }$, it decreases more slowly than $|{A}_{1}\left(z,{t}_{1}\right)|$.
5. With *t*_1_ closed to the peaks of inputs ${A}_{1}\left(z=0,{t}_{1}\right)$ and ${A}_{2}\left(z=0,{t}_{1}\right)$, ${A}_{1}\left(z,{t}_{1}\right){e}^{\frac{1}{2}{ik}{t}_{1}^{2}}$ hits zero along *z* rapidly and turns increasingly negative around *z* = 1 mm, as evidence by the revival of tailing part of $|{A}_{1}\left(z > 1{\rm{mm}},{t}_{1}\right)|$ in Fig. 3(b), while ${A}_{2}\left(z,{t}_{1}\right){e}^{\frac{1}{2}{ik}{\left({t}_{1}-\Delta {t}_{12}\right)}^{2}}{\rm{and}}-{iQ}\left(z,{t}_{1}\right)$ remain positive but both decreasing. This indicates that the Raman process now goes in the reverse direction with ${A}_{2}\left(z,{t}_{1}\right){\rm{and}}-{iQ}\left(z,{t}_{1}\right)$ transferring energy back to ${A}_{1}\left(z,{t}_{1}\right)$ (with a *π* phase shift). Such reversal is characteristic of three-wave mixing or parametric processes^25^. However, with $-{iQ}\left(z,{t}_{1}\right)$ significantly reduced from the maximum, the recovery of $|{A}_{1}\left(z,{t}_{1}\right)|$ is only partial, reaching a peak value much lower than that of the incoming ${A}_{1}\left(z=0,{t}_{1}\right)$ and subsequently decaying away. With low enough ${A}_{1}\left(z=0,{t}_{1}\right)$ and ${A}_{2}\left(z=0,{t}_{1}\right)$ at *t*_1_ away from 0, ${A}_{1}\left(z,{t}_{1}\right)$ does not decay to zero and $-{iQ}\left(z,{t}_{1}\right)$ remains appreciable over the length (L = 2 mm) of the diamond.

The above description is summarized more explicitly in Fig. 3(e), (f) showing plots of evolutions of ${A}_{1}\left(z,{t}_{1}\right){e}^{i\frac{1}{2}k{t}_{1}^{2}}$ and $-{iQ}\left(z,{t}_{1}\right)$ along z for several selected *t*_1_, representing fields from leading (-1.2 ps) to trailing parts ( + 1.2 ps) of the *ω*_1_ pulse, respectively. They are from line cuts of the 3D plots in Fig. 3(b), (d) at the selected ${t}_{1}$ values. Specifically, from Fig. 3(e) or (b), we can obtain the output pulses of ${A}_{1}\left(z=L,{t}_{1}\right)$ and ${|{A}_{1}\left(z=L,{t}_{1}\right)|}^{2}$ for L = 2 mm displayed in Fig. 3(g). Comparison of the latter with the experimental result in Fig. 3(a) exhibits fairly good agreement except for some details towards the end of the output pulse presumably due to deviation of our input parameters in the calculation from the real ones. In Fig. 3(f), we notice that *-iQ* has a broad distribution along *z* at all selected ${t}_{1}$ values, but at ${t}_{1}$ with higher ${A}_{1}\left(z=0,{t}_{1}\right)$ and ${A}_{2}\left(z=0,{t}_{1}\right)$, the stronger Raman interaction leads to a higher and narrower *-iQ* distribution along *z* with its maximum appearing at smaller *z*. At ${t}_{1} \sim 0.6\,{\rm{ps}}$, the integration of *-iQ* over the length of the diamond from *z* = 0 to 2 mm, i.e., area under the curve of *-iQ* versus *z* in Fig. 3(f), is close to or higher than those at other ${t}_{1}$, and therefore, following Eq. (6), if the fs MIR pulse comes in to beat with *-iQ* at ${t}_{1} \sim 0.6\,{\rm{ps}}$, the THz output is close to maximum.

## Optimization of THz output via Phonon Amplitude *Q*(*z,t*_1_) control

**Fig. 4:**
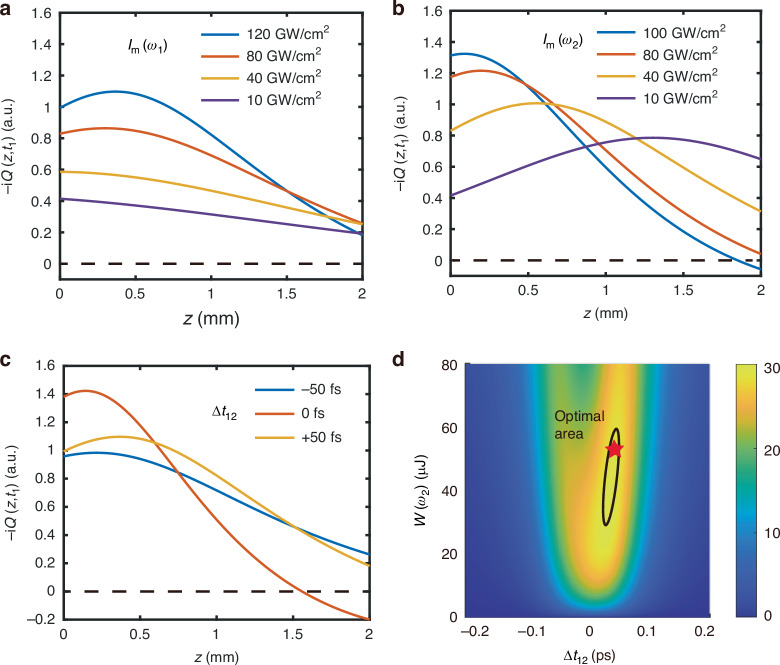
Optimizaion of THz output through phonon amplitude control. Calculated distributions of $-{iQ}\left(z,{t}_{1,{\rm{MIR}}}\left(z\right)\right)$ along the length of the 2-mm diamond with ${t}_{1,{\rm{MIR}}}\left(z\right)\approx -0.6{\rm{ps}}$ for **a** different values of *I*_*m*_(*ω*_1_) with *I*_*m*_(*ω*_2_) fixed at 55 GW cm^−2^ and $\Delta {{\rm{t}}}_{12}$ at 50 fs, **b** different values of *I*_*m*_(*ω*_2_) with *I*_*m*_(*ω*_1_) fixed at 110 GW cm^*-*^_2_ and $\Delta {{\rm{t}}}_{12}$ at 50 fs, and **c** $\Delta {{\rm{t}}}_{12}=0{\rm{and\; \pm }}50{\rm{fs}}$ with *I*_*m*_(*ω*_1_) with *I*_*m*_(*ω*_2_) fixed at 110 GW cm^−2^ and 55 GW cm^−2^, respectively. The horizontal dash lines denote -i*Q*(*z*) = 0. **d** A 3D plot describing the dependence of the THz output energy, $W\left({\omega }_{{\rm{THz}}}\right)$, on $W({\omega }_{2})$ and $\Delta {{\rm{t}}}_{12}$. The red star labels the values of $W({\omega }_{2})$ and $\Delta {{\rm{t}}}_{12}$ used in our experiment

We now discuss how *Q*(*z,t*_1_), and hence the THz output, will vary with input parameters ${I}_{m}\left({\omega }_{1}\right)$, ${I}_{m}\left({\omega }_{2}\right)$, and $\Delta {t}_{12}$. We focus on the evolution of *Q*(*z,t*_1_) at *t*_1_ = 0.6 ps, at which the fs MIR pulse comes in to beat with *Q* can generate near-maximum THz output (*t*_1,MIR_ = 0.6 ps). Displayed in Fig. 4(a-c) are numerical calculation results on -i*Q*(*z,t*_1_) versus *z* for three different cases: (a) *I*_*m*_(*ω*_2_) = 55 GW cm^-2^, $\Delta {{\rm{t}}}_{12}=50\,{\rm{fs}}$, and a set of different values of ${I}_{m}\left({\omega }_{1}\right)$, (b) ${I}_{m}\left({\omega }_{1}\right)=110\,{\rm{GW}}{{\rm{cm}}}^{-2}$, $\Delta {t}_{12}=50\,{\rm{fs}}$, and a set of different values of ${I}_{m}\left({\omega }_{2}\right)$, and (c)${I}_{m}\left({\omega }_{1}\right)=110\,{\rm{GW}}{{\rm{cm}}}^{-2}$, ${I}_{m}\left({\omega }_{2}\right)=55\,{\rm{GW}}{{\rm{cm}}}^{-2}$, and three different $\Delta {t}_{12}$. The corresponding THz output fluence versus ${I}_{m}\left({\omega }_{1}\right)$, ${I}_{m}\left({\omega }_{2}\right)$ and $\Delta {t}_{12}$ are shown in SI Sec. 9. They provide a clear picture on how to adjust the three input parameters to optimize the THz output. Most importantly, as indicated in Eq. (5), we would like to have ${\int }_{0}^{L}-{iQ}(z,{t}_{1}){dz}$ with *t*_1_ = 0.6 ps (i.e., the area under the curves in Fig. 4(a-c)) be as large as possible. This means that we should have a distribution of -i*Q*(*z,t*_1_) with a broad maximum that is as high as possible over the whole length of the crystal.

From our numerical calculations presented in Figs. 3 and 4, we learn that stronger transient Raman excitation creates a higher maximum of -i*Q*(*z,t*_1_), but depletes the *A*_1_ input faster and results in a narrower distribution of -i*Q*(*z,t*_1_) along *z*. Reducing the Raman excitation suppresses the peak of -i*Q*(*z,t*_1_), but broadens the distribution of -i*Q*(*z,t*_1_). The ideal situation for maximum THz output is somewhere in the middle, a broad -i*Q*(*z,t*_1_) maximum as large as possible and covering roughly the whole diamond crystal length. As seen from Fig. 4a, b, we should have ${I}_{m}\left({\omega }_{1}\right)$ as large as possible. We then adjust ${I}_{m}\left({\omega }_{2}\right)$ accordingly to tune the distribution of -i*Q*(*z,t*_1_) along *z* with ${N}_{2}\left(z\right) \sim {N}_{1}(z)$ appearing around the middle of the crystal length and maximizing ${\int }_{0}^{L}-{iQ}(z,{t}_{1}){dz}$. In practice, ${I}_{m}\left({\omega }_{1}\right)$ is limited by the optical damage threshold of diamond. In our case referring to Fig. 4a, b, with ${I}_{m}\left({\omega }_{1}\right)=110\,{\rm{GW}}{{\rm{cm}}}^{-2}$ (80% of the optical damage threshold) and $\Delta {{\rm{t}}}_{12}=50\,{\rm{fs}}$, the maximum of ${\int }_{0}^{L}-{iQ}(z,{t}_{1}){dz}$ happens at ${I}_{m}\left({\omega }_{2}\right) \sim 30$ GW cm^−2^ (see SI Sec. 9 Fig. S8(b)). With ${I}_{m}\left({\omega }_{1}\right)$ fixed, lowering ${I}_{m}\left({\omega }_{2}\right)$ is to reduce the Raman excitation and broaden the distribution of $-{iQ}\left(z,{t}_{1}\right)$.

Another way of reducing the Raman excitation is to detune it from resonance. As mentioned earlier, setting $\Delta {t}_{12}$ away from 0 is equivalent to detuning Raman excitation. At ${I}_{m}\left({\omega }_{2}\right) \sim 55$ GW cm^−2^ with ${I}_{m}\left({\omega }_{1}\right) \sim 110$ GW cm^−2^, the resonant Raman excitation (with $\Delta {{\rm{t}}}_{12}=0)$ is too strong, yielding a $-{iQ}(z,{t}_{1})$ distribution too narrow. To reduce the excitation, we need to lower ${I}_{m}\left({\omega }_{2}\right)$ or detune it from resonance. In Fig. 4c, it is seen that setting $\Delta {{\rm{t}}}_{12}={\rm{\pm }}50\,{\rm{fs}}$ visibly improves the distribution of -i*Q*(*z,t*_1_) and enhances ${\int }_{0}^{L}-{iQ}(z,{t}_{1}){dz}$. We note that the curves for $\Delta {{\rm{t}}}_{12}=+50\,{\rm{fs}}$ and -50 fs are somewhat different. This is because we have included the group velocity difference of the ${\omega }_{1}$ and ${\omega }_{2}$ pulses in diamond in the calculation.

To find the total THz output, we extend our numerical calculation over the whole beam overlapping area. With input ${A}_{1}\left(z=0,\vec{{\rho }},{t}_{1}\right)$ and ${A}_{2}\left(z=0,{\vec{{\rho }},t}_{1}\right)$ known from the spatial profile of the beam intensity dependence on $\vec{{\rho }}$, we calculate $-{iQ}(z,\vec{{\rho }},{t}_{1})$. and ${A}_{\rm{THz}}(z=L,\vec{{\rho }},{t}_{1})$, and then integration of ${|{A}_{\rm{THz}}(z=L,\vec{{\rho }},{t}_{1})|}^{2}$ over $\vec{{\rho }}$ and ${t}_{1}$ gives the THz output energy per pulse. As one would expect, such an integration decreases the THz output per unit area compared to that from the central beam area. The results of THz output versus $W\left({\omega }_{1}\right),W\left({\omega }_{2}\right)$, $W\left({\omega }_{\rm{THz}}\right)$, and $\Delta {t}_{12}$ are depicted in Fig. 2(c-f) in comparison with experiment. The agreement is fairly good. Particularly, Fig. 2(d) shows that with $W\left({\omega }_{1}\right)=64\,{\rm{\mu}}{\rm{J}}\,{\rm{and}}\,\Delta {t}_{12}=50\,{\rm{fs}}$ fixed, the THz output appears to approach saturation with increase of $W\left({\omega }_{2}\right)$ towards 55 μJ, but as described earlier, the THz output actually goes through a broad maximum centered around $W\left({\omega }_{2}\right)=55\,{\rm{\mu }}{\rm{J}}$; it would drop if $W\left({\omega }_{2}\right)$ is further increased. We note that, the experimental THz output with $W\left({\omega }_{2}\right) < 20\,{\rm{\mu }}{\rm{J}}$ is larger than the theoretical curve with $\Delta {t}_{12}=50\,{\rm{fs}}$. The reason is that in adjusting the beam geometry in our experiment to maximize the THz output, we must have inadvertently reduced $\Delta {t}_{12}$ to near zero, which is more appropriate for maximizing THz output with low $W\left({\omega }_{2}\right)$. As seen in Fig. 2(d), the theoretical curve with $\Delta {t}_{12}=0$ fits the data for $W\left({\omega }_{2}\right) < 20\,{\rm{\mu }}{\rm{J}}$ quite well. In Fig. 2(f) with $W\left({\omega }_{1}\right)=64\,{\rm{\mu }}{\rm{J}}$ and $W\left({\omega }_{2}\right)=55\,{\rm{\mu }}{\rm{J}}$ fixed, it is seen that the THz output versus $\Delta {t}_{12}$ exhibits two asymmetric peaks at $\Delta {t}_{12}={\rm{\pm }}50\,{\rm{fs}}$ as mentioned in earlier discussion. With $W\left({\omega }_{1}\right)=64\,{\rm{\mu }}{\rm{J}}$ and $W\left({\omega }_{2}\right)=5\,{\rm{\mu }}{\rm{J}}$, the THz output versus $\Delta {t}_{12}$ has only a single peak at $\Delta {t}_{12} \sim 0$. To see more details on how the THz output, pumped by $W\left({\omega }_{1}\right)=64\,{\rm{\mu }}{\rm{J}}$, varies with $W\left({\omega }_{2}\right)$ and $\Delta {t}_{12}$, we display our calculated results in a 3D plot in Fig. 4(d). The theoretical curves in Figs. 2(d), (f) were obtained from vertical line cuts at $\Delta {t}_{12}=50\,{\rm{fs}}$ and 0 fs, and horizontal line cuts at $W\left({\omega }_{2}\right)=55\,{\rm{\mu }}{\rm{J}}$ and 5 μJ, respectively, of the 3D plots. We notice from Fig. 4(d) that the THz output has a broad hump around the maximum in the 2D phase space; the narrow stripe encircled by the black line specifies a region where the THz output is within 5% of the maximum. The star on the 3D plot marks the point of $W\left({\omega }_{2}\right)$ and $\Delta {t}_{12}$ used in our experiment. It is seen that the THz output is indeed near maximum. We note that with $W\left({\omega }_{2}\right)\,{\rm{at}}\,20\,{\rm{\mu }}{\rm{J}}$ and $\Delta {t}_{12}$ at 10 fs, the THz output is still within 10% of the maximum.

## Optimization of THz output from Longer Diamond

**Fig. 5:**
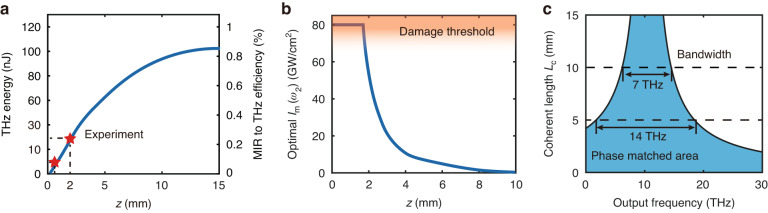
Calculation of THz output from longer diamond. **a** Calculated optimal THz output energy (left axis) and the corresponding MIR to THz conversion efficiency (right axis) versus the crystal length. Here, the peak intensity of *ω*_1_ at the beam center *I*_*m*_(*ω*_1_) = 110 GW cm^−2^, $\Delta {t}_{12}=50{\rm{fs}}$, $\Delta {t}_{1,{\rm{MIR}}}=0.6{\rm{ps}}$, $W\left({\omega }_{{\rm{MIR}}}\right)=14{\rm{\mu }}{\rm{J}}$ and *I*_*m*_(*ω*_2_) is optimized for each crystal length *L*. The beam sizes are similar to the experimental values. The red star denotes the experimental result (*L* = 2 mm*, W*(*ω*_THz_) = 30 nJ, *L* = 0.5 mm, *W*(*ω*_THz_) = 10 nJ). **b** Numerically predicted optimal *I*_*m*_(*ω*_2_) versus crystal length. The *I*_*m*_(*ω*_1_) used for calculation in (a-c) is fixed at 110 GW cm^−2^ (64 μJ per pulse). **c** Coherence length, ${L}_{c}\left({\omega }_{{\rm{THz}}}\right)=\pi /|\Delta k\left({\omega }_{{\rm{THz}}}\right)|$, versus THz frequency, where $\Delta k\left({\omega }_{{\rm{THz}}}\right)$ is the phase mismatch. The strain is optimized for perfect phase match at 10 THz

We can now proceed to discuss how the THz output can be enhanced with longer diamond, knowing that it is currently possible to fabricate synthetic diamond crystals over 1 cm thick by CVD. It is still true that *I*_*m*_(*ω*_1_) should be as large as possible for all crystal lengths, but is limited by optical damage in practice. (see SI section 10). In the assessment below, we set *I*_*m*_(*ω*_1_) = 110 GW cm^−2^, which is 80% of the damage threshold (140 GW cm^−2^) for diamond. Then again, for a given diamond length, we could calculate the dependence of THz output on *I*_*m*_(*ω*_2_), $\Delta {t}_{12}$ and $\Delta {t}_{1,{\rm{MIR}}}$ like the case of *L* = 2 mm shown in Fig. 4d. The calculation for *L* > 2 mm is more complicated because the difference of the group velocities at *ω*_1_ and *ω*_2_ and the phases of ${A}_{1}{e}^{\frac{1}{2}{ik}{t}_{1}^{2}},{A}_{2}{e}^{\frac{1}{2}{ik}{\left({t}_{1}-\Delta {t}_{12}\right)}^{2}}$, and -*iQ* are no longer negligible. The physical picture described earlier on how the THz output gets optimized is however still the same. From the calculation, we realized that for optimization of the THz output, *I*_*m*_(*ω*_2_) was the most relevant adjustable input parameter, while for all crystal lengths, $\Delta {t}_{12}$ and $\Delta {t}_{1,{\rm{MIR}}}$ could be set at 50 fs and 0.6 ps, respectively, see SI section 11. The calculated optimum THz output versus crystal length *L* is presented in Fig. 5a and the corresponding *I*_*m*_(*ω*_2_) versus *L* in Fig. 5b. The experimentally measured THz outputs at *L* = 0.5 and 2 mm are marked by the red stars in Fig. 5a. We notice that the THz output energy only gets enhanced by ~2 times with increase of L from 2 to 5 mm and further enhanced to 1.5 times with *L* increased to 10 mm. In practice, it would be increasingly much more difficult to get diamond of longer length and THz diffraction may set in if the beam overlapping area in diamond is small. Therefore, for THz generation from diamond, *L* < 5 mm seems to be close to ideal.

It is known that for phase-matched nonlinear wave mixing in a medium, the output band width is given by $|\Delta k\left({\omega }_{\mathrm{THz}}\right){|L}\le \pi$ if the medium length *L* is smaller than π/|*∆k|* with |*∆k|* being the phase mismatch along *L*. A longer medium leads to a narrower output band width and a broader output pulse width. For our case of CSRS in strained diamond with *∆k* = 0 at 10 THz, we show in Fig. 5d the calculated coherence length versus the output THz frequency. The output THz bandwidth for a given diamond length *L* can be obtained from a horizontal line cut at L in the figure. For *L* = 5 and 10 mm, the bandwidths are 14 and 7 THz, corresponding to 31.5 and 63 fs, respectively.

## Conclusion and Perspective

We have experimentally demonstrated that collinear phase matching of CSRS for generation of gapless, tunable, fs, THz pulses can be achieved in properly strained diamond. Using a commercial 5-W fs laser system with associated OPA/DFG stages, we were able to generate 5-15 THz, 60-fs THz pulses with 30 nJ per pulse at 10 THz, focusing of which to an area of ~π(55 × 75)/2 μm^2^ yielding a peak field strength of ~2.3 MV cm^−1^. From the perspective of system integration, the collinear phase-matching geometry adopted in our CSRS-based THz source reduces alignment complexity and improves compatibility with commercial ultrafast laser systems. Unlike non-collinear configurations, which require precise angular alignment and often lead to spatio-temporal distortion of the THz beam, the collinear geometry provides greater tolerance to beam alignment. This configuration preserves the spatial and temporal quality of the THz radiation and is readily compatible with standard THz time-domain spectroscopy setups and other ultrafast spectroscopic techniques.

Theoretical calculation based on solving a set of coupled wave equations that describe transient CSRS has been worked out to well explain the experimental results. It provides a clear physical picture on how the two input pulses excite a phonon distribution in time and space in diamond through transient coherent Raman excitation, followed by beating of a fs mid-infrared pulse to beat with the phonons to generate a high-quality fs THz pulse. From the physical picture and numerical calculation, we learn how to optimize the THz output energy by adjusting the input parameters, i.e., intensities of three input pulses and time separations among them. The main point is that the integration of the phonon distribution along the length of the diamond should be nearly maximized. This requires the pump beam to be as intense as possible (limited by optical damage) and the Stokes input beam to have an appropriate intensity for Raman excitation to yield the desired phonon distribution in diamond. Too strong Stokes intensity narrows the phonon distribution too much, and too weak intensity broadens the distribution too much.

With high THz output, the pump depletion is strong even in thin films of diamond ( ~ 0.5 mm), and the phase-matched THz output from diamond increases sub-linearly with diamond length. With the length increasing from 0.5 to 2, to 5, and to 10 mm, the THz output ratio is expected to increase roughly from 1 to 3 to 6 and to 9. The optimized 60-fs output pulse at 10 THz from a 5-mm diamond with a beam overlapping area of *π*(150×150)μm^2^/2 would reach ~60 nJ per pulse in our setup; focusing the output to a near diffraction-limited spot would yield a peak field strength of 3.2 MV cm^−1^. Since diamonds thicker than 5 mm are currently not easily available, such an output is near the limit of our CSRS scheme for THz generation. Further increase of the output pulse energy, we need a more powerful fs laser system to boost the energies of the three input pulses. The THz output is directly proportional to the energy of the fs mid-IR pulse, which is limited by its optical damage threshold (7 TW cm^−2^)^20^, but it is still one order of magnitude larger than the current experimental value (0.7 TW cm^−2^). Higher input pump and Stokes pulse energies would allow us to increase the beam overlapping area in diamond and the THz output energy is proportional to the area. It is also possible to arrange to pass multiple fs mid-IR pulses through diamond to generate multiple fs THz pulses.

## Supplementary information

Supplementary information for “Gapless Tunable Intense Terahertz Pulse Generation in Strained Diamond”

## Data Availability

All data are available in the main text or the supplementary materials.
