# Supplementary material for: Gapless tunable intense terahertz pulse generation in strained diamond
Source: Light Sci Appl. 2026 Mar 31;15:186. doi: 10.1038/s41377-025-02092-6 (PMC13039125; doi:10.1038/s41377-025-02092-6)
Supplement: Supplementary file 1 — Supplementary information for “Gapless Tunable Intense Terahertz Pulse Generation in Strained Diamond” [file 41377_2025_2092_MOESM1_ESM.docx]

**Supplementary information for “Gapless Tunable Intense Terahertz Pulse Generation in Strained Diamond”**

**Authors:** Yudan Su1,2,3,4, Yuxuan Wei1,4, Chaonan Lin4,5,6, Li Huang1, Jiaming Le1, Chong-xin Shan5,6, A. H. Kung1, Chuanshan Tian1*, Y. Ron Shen1,2*

**Affiliations:**

1Department of Physics, State Key Laboratory of Surface Physics and Key Laboratory of Micro- and Nano-Photonic Structure (MOE), Fudan University, Shanghai 200433, China.

2 Department of Physics, University of California, Berkeley, California 94720, USA.

3 Zhangjiang Laboratory, 100 Haike Road, Shanghai 201204, China

4 These authors contributed equally to this work.

5 Henan Key Laboratory of Diamond Optoelectronic Materials and Devices, School of Physics and Microelectronics, Zhengzhou University, Zhengzhou, 450052, China

6 Institute of Quantum Materials and Physics, Henan Academy of Sciences, Zhengzhou 450046, China

*Corresponding authors. Emails: [yrshen@berkeley.edu](mailto:yrshen@berkeley.edu); cstian@fudan.edu.cn

1. **Input Parameters in Eq(1a-d)**

The transient phonon-photon coupling wave equations are given in Eq(1a-c) in the main text. The parameters, and represent the Raman tensor and damping rate of the phonon, respectively. The Raman tensor can be determined using the known nonlinear coefficient =1.4×10-19 m2 V-2 for the resonant stimulated Raman scattering process1. The refractive indices of the *ω*1, *ω*2 and THz are , respectively. is the group velocity and MIR group velocity in Eq (5) is . denotes the vacuum dielectric constant. The slowly varying amplitude approximation is employed, and the group velocity of the Raman phonon wave is neglected. Additionally, the contribution of the coherent stokes scattering of MIR pulse as well as the depletion of MIR is neglected due to the low conversion efficiency and we assume the collinear phase matching condition is achieved with after applying the strain.

The input peak electric fields at the beam center is calculated from , where , where are provided in the main text and is the transmittance. The MIR to THz conversion efficiency is calculated by: .

1. **Explicit expression of Eq (1d).**

The slowly varying approximation in time in Eq. (1d) of the main text breaks down if the THz bandwidth is close to or larger than the central frequency. However, the problem can be avoided if one first deals with the frequency components of and later Fourier-transform them back to the time domain. In this treatment, the wave equation for the component of is

and the slowly varying approximation in *z* gives with . The THz output energy is proportional to , where is the spectrum of the input MIR pulse amplitude. The slowly varying approximation in time is to replace in the integral by . Thus, the correction factor for not taking the approximation is

If the MIR spectrum can be approximated by a Gaussian , we have . For , we find *K* = 1.1 and 1.4 at THz and 5 THz, respectively.

1. **Experimental setup**

The experimental arrangement is depicted in Fig. S1. A commercial 33 fs, 5.2 W, 1 kHz Ti:sapphire laser system with a center wavelength of 808 nm was used to pump two optical parametric amplifiers (OPA) seeded by a common white light. The signal 1 (*ω*1) and idler 1 (*ω*2) pulse from OPA1 were 47 fs pulses tuned to frequencies of *ω*1/2*π*= 206 THz and *ω*2/2*π*= 166 THz, respectively, and were then chirped into picosecond pulses by two ZnSe rods with optical path lengths of 50 mm and 67 mm. The instantaneous beat frequency of the two pulses was fixed to match the Raman phonon frequency *ω*r/2*π*= 40 THz. Meanwhile, The MIR pulses, with the center frequency tunable from 45 to 60 THz and a duration of 63 fs, were derived from a difference frequency generation crystal (GaSe), pumped by the signal 2 and idler 2 from OPA2. The chirped *ω*1 and *ω*2 beams were firstly combined by a dichroic mirror and then combined with the MIR pulse by a long-pass filter. Three beams were collinearly focused onto a 2 mm × 2 mm × 2 mm diamond cube synthesized by MPCVD technique. The cube was mounted between two steel blocks in a homemade strain machine2. To ensure high parallelism, the diamond was manually polished and the polishing angle was adjusted based on the testing results of the photoelectric collimator with a resolution of . The geometry of the beam polarizations and crystal axis is illustrated in Fig. 1b in the main text. The maximum pulse energies of the *ω*1, *ω*2, and MIR pulses in the diamond are 64 μJ, 55 μJ, and 14 μJ, respectively. The beam areas of *ω*1, *ω*2, and MIR pulse are *π*(185×164) μm2/2, *π*(236×215) μm2/2 and *π*(139×117) μm2/2, respectively. The rest of the pulses passed through a centered hole on the first parabolic mirror and were blocked by a damper. The output THz pulse from the diamond was refocused into a Far-IR power meter (BL-THz, Gentec) or directed to a homemade Fourier Transform Infrared Spectrometer (FTIR) to obtain its spectra. Here, we inserted a 1.5 mm thick Polyethylene (PE) filter into the THz beam path to prevent scattered MIR background from interfering with the measurement of the power and spectra of THz pulses.


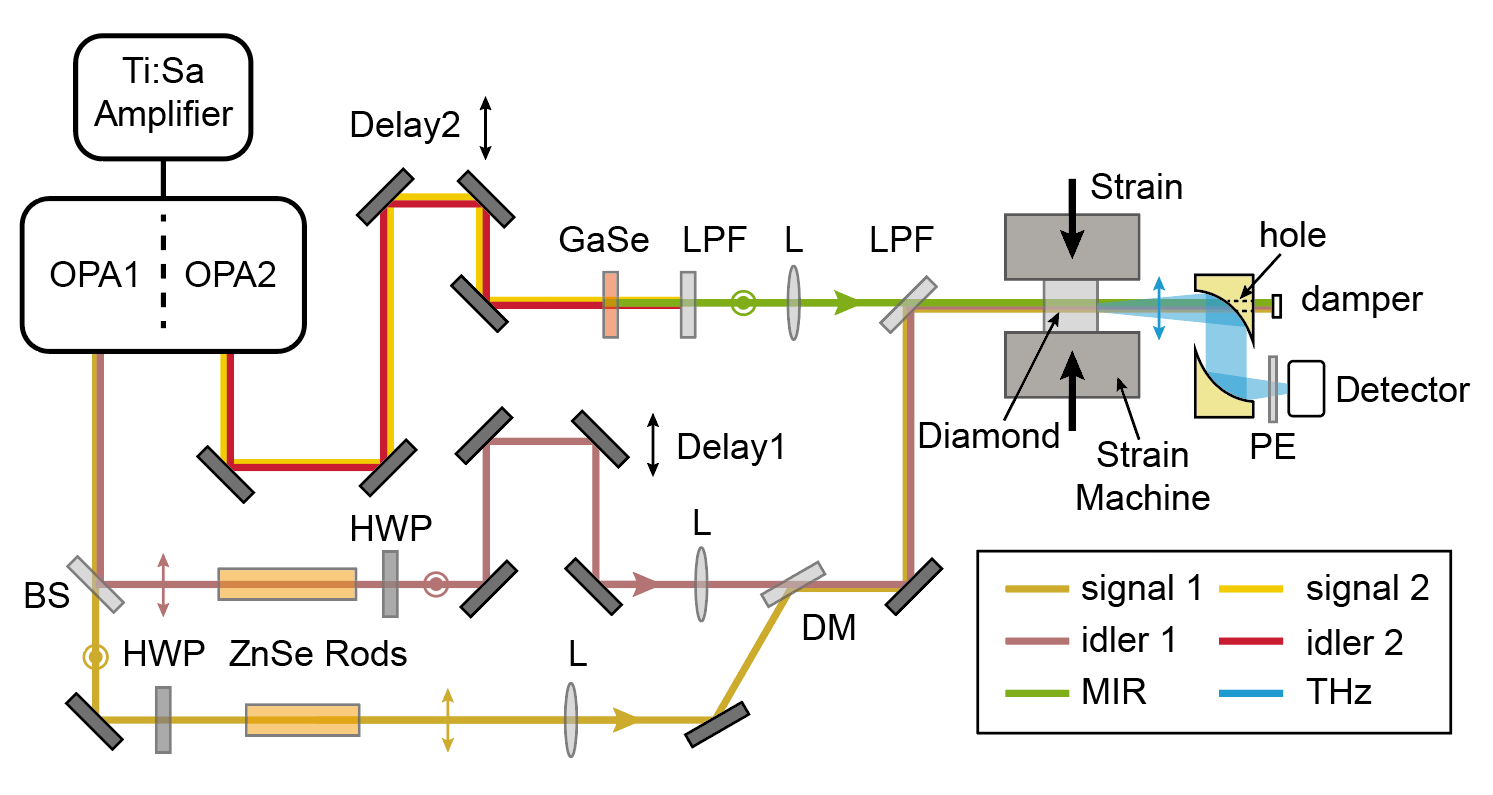


Fig. S The sketch of the collinear R-FWM setup.BS (Beam splitter), DM (Dichroic mirror), LPF (Long pass filter), L (Lens), HWP (Half wave plate).

1. **Characterizing temporal profile and chirp of the *ω*1 and *ω*2 beams**

The temporal profiles of the *ω*1 and *ω*2 pulses were measured by cross-correlation technique, as depicted in Fig. S2a. The *ω*1 or *ω*2 pulse was overlapped with a 33 fs 800 nm gate pulse on a BBO crystal. The sum frequency generation (SFG) signal was then measured by a photodiode at a different time delay of the gate pulses. The temporal profiles of the *ω*1 and *ω*2 are shown in Fig. S2b, while the depleted *ω*1 profile after passing through the diamond crystal is presented in Fig. 3a in the main text. Although in the theoretical calculation, we approximate two pulses as Gaussian pulse, it doesn’t influence the THz output because phonon amplitude *Q*(*z,t*) at *t*1,MIR is proportional to the integration of over time, given by Eq(1a)

If we switch the photodiode to a spectrometer to detect the SFG signal, the transient frequency spectra of two chirped pulses can be obtained, as shown in Fig. S2c. The chirp rates of the incident *ω*1 and *ω*2, determined by linear fitting by the peak frequency of the SFG signal over time delay, are 7.0±0.1 THz ps-1, respectively.


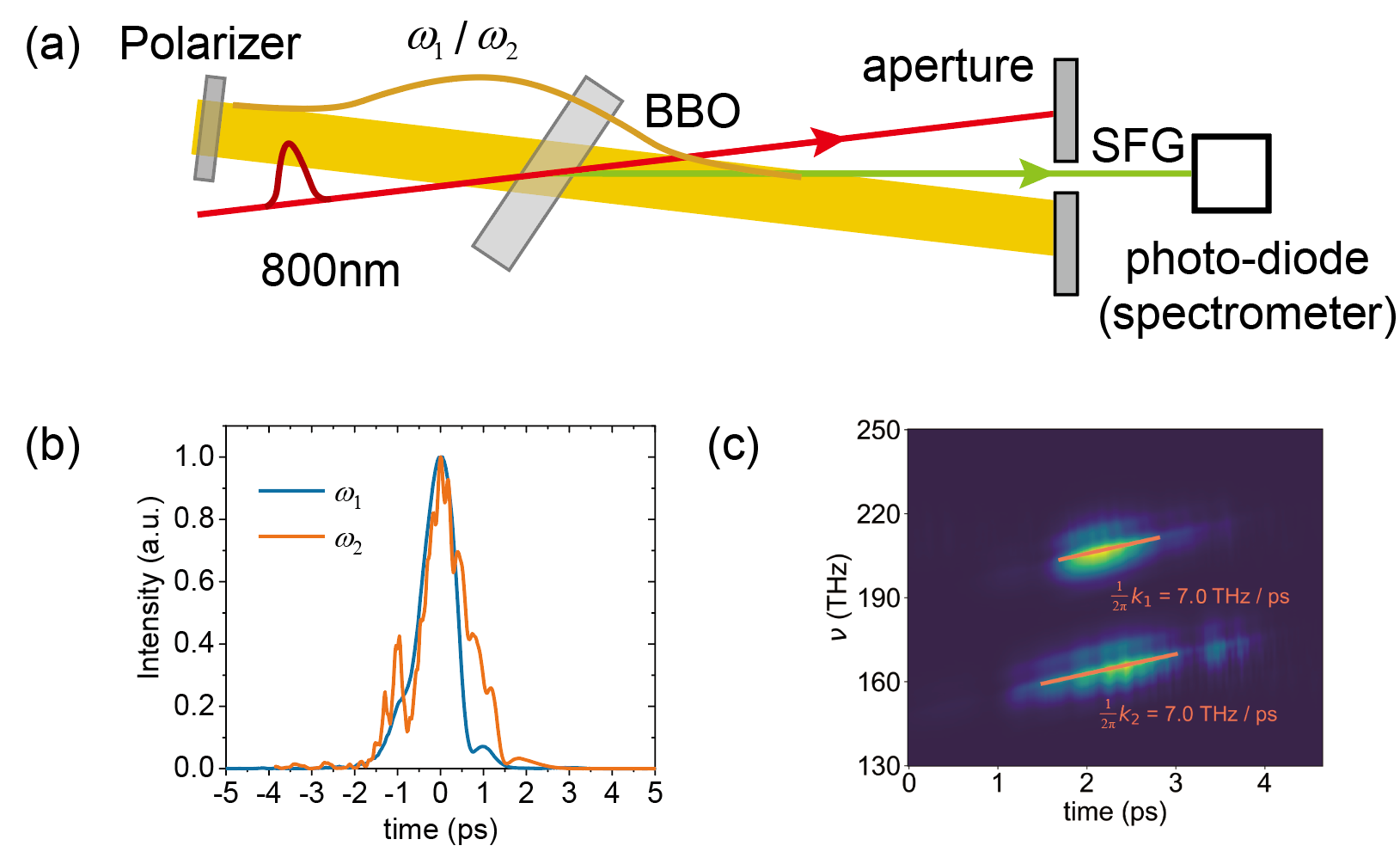


Fig. S (a) The sketch of the intensity cross-correlation setup. (b) times profiles of the *ω*1 and *ω*2 pulses. Some oscillations can be obtained in the time profiles of the *ω*2 pulses, which are induced by the interference from the coating on the LPF beam combiner in Fig. S1a. (c) The transient frequency spectra of *ω*1 and *ω*2 pulses

1. **The optimization and calibration of the strain**
2. Elastic-optical effect and optimized strain

When a uniaxial strain is applied along [001] axis of diamond, the dielectric tensor variation is given by3:

Here, is the bulk dielectric constant, *a* is the lattice constant, represents the strain. The photoelastic constants are .3 The polarizations of the *ω*1 and THz (*ω*THz) pulses are along [001] direction, while the *ω*2 and MIR (*ω*MIR) pulses are polarized along [110] axis. The changes of dielectric constant for these four beams are given by:

To achieve the collinear phase matching condition , the optimal strain is found to be -0.13% for generating 10 THz output. The variation of the optimal strain for different THz center frequencies is illustrated in Fig. 1c in the main text. The output THz energy generated by a diamond with a thickness of *L* under a uniform strain field of is proportional to as shown in Fig. 1d in the main text.

1. Strain distribution and the influence of strain uniformity on THz generation

We employed the orthogonal polarization method to image the strain distribution in the diamond. As sketched in Fig. S3a, the 633 nm laser beam from a He-Ne laser is initially polarized at 45° relative to the [001] axis by polarizer 1 (P1), while the polarization of the second polarizer (P2) is orthogonal to the first one. When a uniaxial strain is applied, the diamond becomes birefringent. The linear polarized light becomes elliptical after passing through the diamond. Such birefringence can be recorded using CCD camera after P2. The strain distribution in the x-y plane is shown in Fig. S3b with the pump beam path marked by a white dashed circle. The strain difference between two adjacent bright stripes is approximately (0.1%)/6 for 633 nm. As a result, the strain can be regarded as uniform for the FWM process. The strain inhomogeneity in the *x*-*z* plane is also plotted in Fig. S3c, with the beam path along the *z*-axis represented by a white dashed line. Achieving homogenous strain distribution in diamond cube is extremely challenging in experiment. Due to the exceptional hardness of diamond relative to most materials, mechanical contact between the diamond and the strain application apparatus inevitably introduces deformation at the interface, leading to inhomogeneous strain fields. In the experiment we monitor the strain distribution by in situ imaging the birefringence pattern in diamond during strain application. This allows us to ensure that the resulting strain profile maintains an acceptable degree of homogeneity (less than 6 strain fringes along the beam path) for efficient THz generation, as illustrated in Fig. S3c)

To investigate the influence of the strain inhomogeneity along the beam path on the THz output, we consider a strain distribution following a parabolic profile, , where is strain at the crystal center, *ns* is numbers of the interference stripes from the center of the diamond to the edge. According to stripe distribution in Fig. S3c, *ns* is about 3 in the experiment. Under undepleted pump approximation, the output THz electric field is proportional to . The THz energy versus strain for 2 mm thick diamond of different *ns* is shown in Fig. S3d. As a result, the decrease of THz energy caused by the strain inhomogeneity is less than 10% for *ns* = 3, which can be considered negligible.


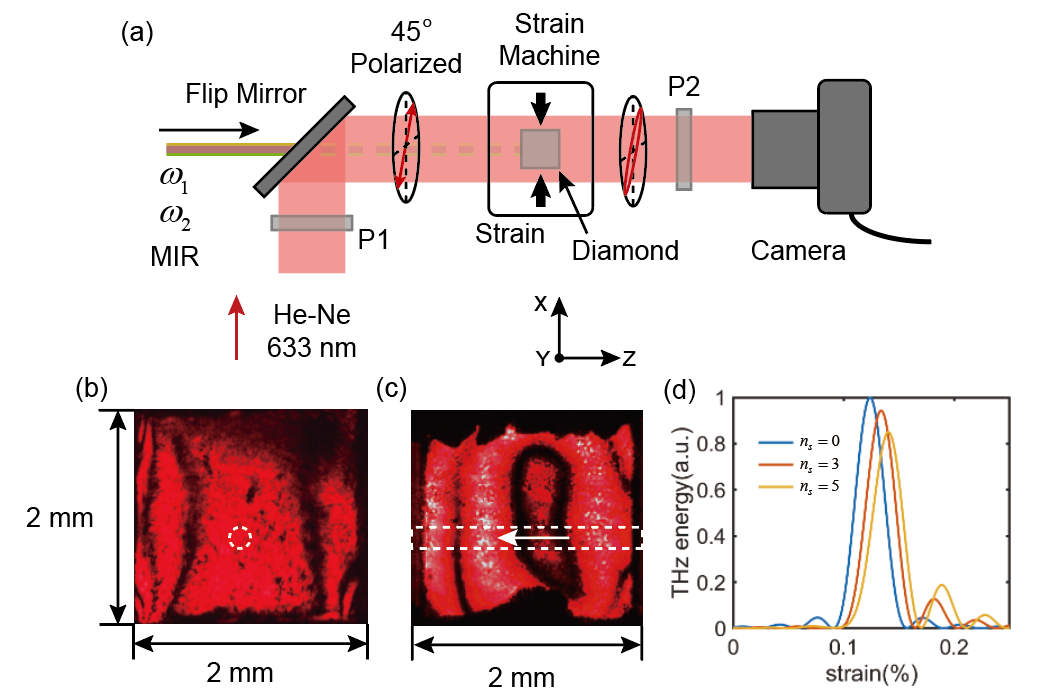


Fig. S (a) The setup of the strain distribution image system using the orthogonal polarization method. The flip mirror is used to switch between the beam paths for THz generation and strain imaging, allowing for in-situ adjustment of the pump lights’ position on the diamond. The strain distribution in the *x*-*y* plane (b) and *y*-*z* plane (c). (d) The influence of strain inhomogeneity on the THz output.

1. **THz output versus the for *L* = 2 mm.**


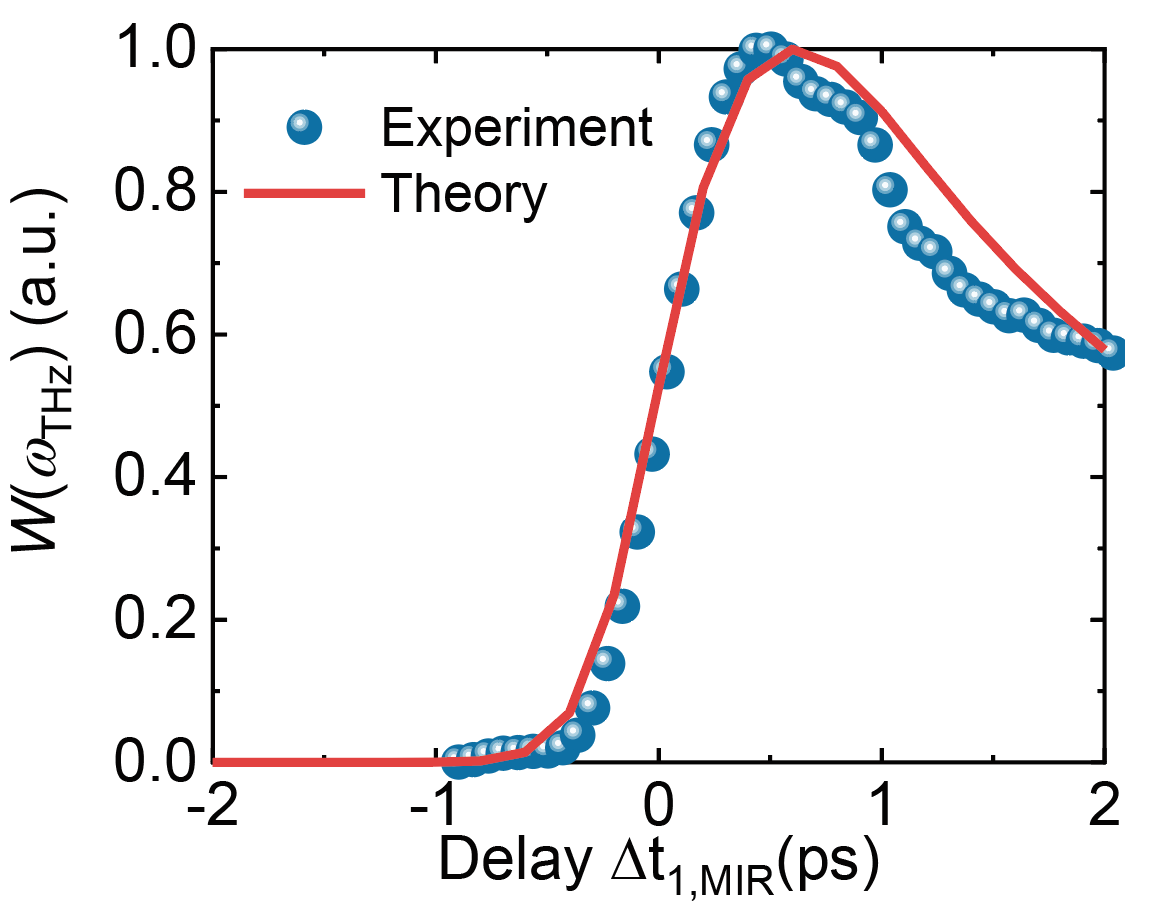


Fig. S Experimental (blue dot) and numerical (red solid curve) THz output versus the for *L* = 2 mm.

1. **THz spatial beam profiles**


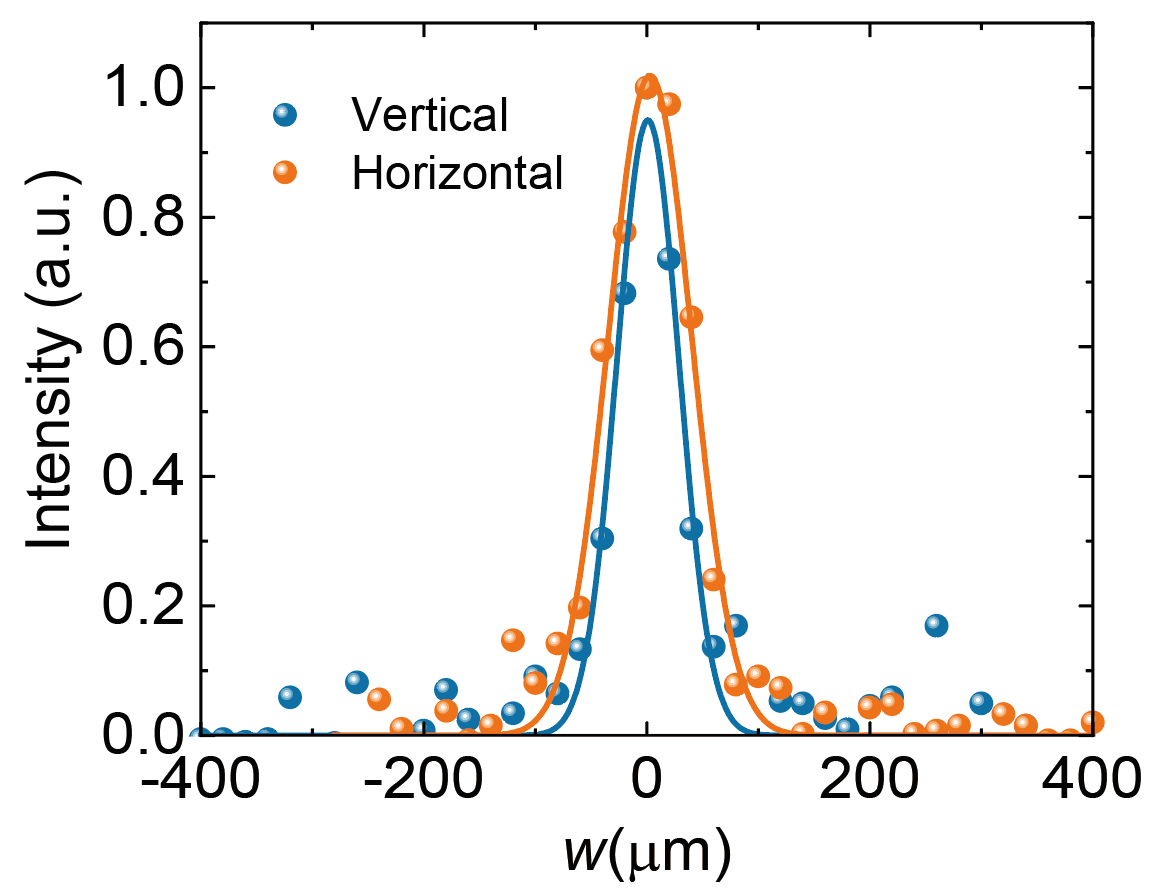


Fig. S THz spatial beam profiles after 2:1 imaging by a pair of off-axis parabolic mirrors. The Gaussian fitting (solid curves) gives the THz beam size of (55◊75)/2 m2 (e-2 radius),

1. **The phase of and**

According to Eq(7a-c) in the main text, if we neglect group velocity mismatch (GVM) term and set , and -*iQ* are then perfect real number. The real, imaginary and absolute values of with 110 GW cm-2,55 GW cm-2, 0 fs and neglecting GVM term, are shown in Fig. S6a. The imaginary value is 0 as expected. After considering GVM and non-zero , they become complex as shown in Fig. S6b. Note that when z > 1.5 mm, the real part of can be comparable with its imaginary part. The real part contribution to the THz output can be neglected because the THz electric field variation for neglecting the imaginary part, , for *L* = 2 mm. and 93.44 % for *L* = 4 mm.


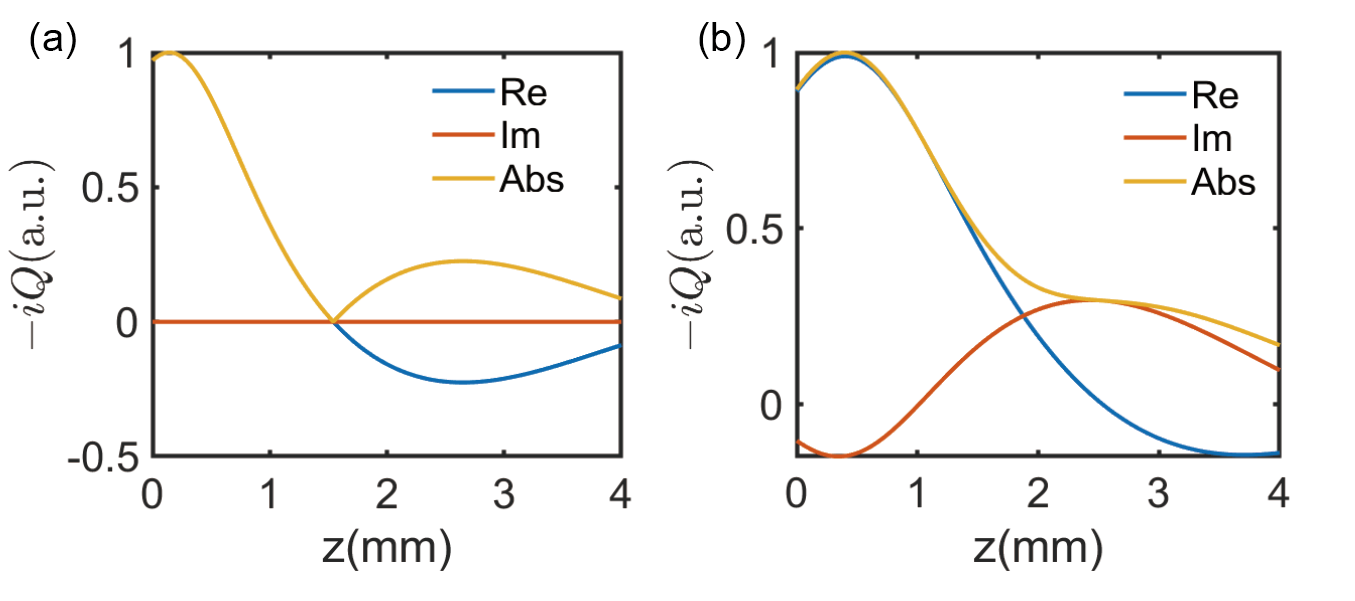


Fig. S The numerical real, imaginary and absolute values of with 110 GW cm-2,55 GW cm-2, (a) 0 fs, neglecting GVM (b) 50 fs, considering GVM.

1. **The THz fluence dependence on the pump intensity and the time delay at the beam center**


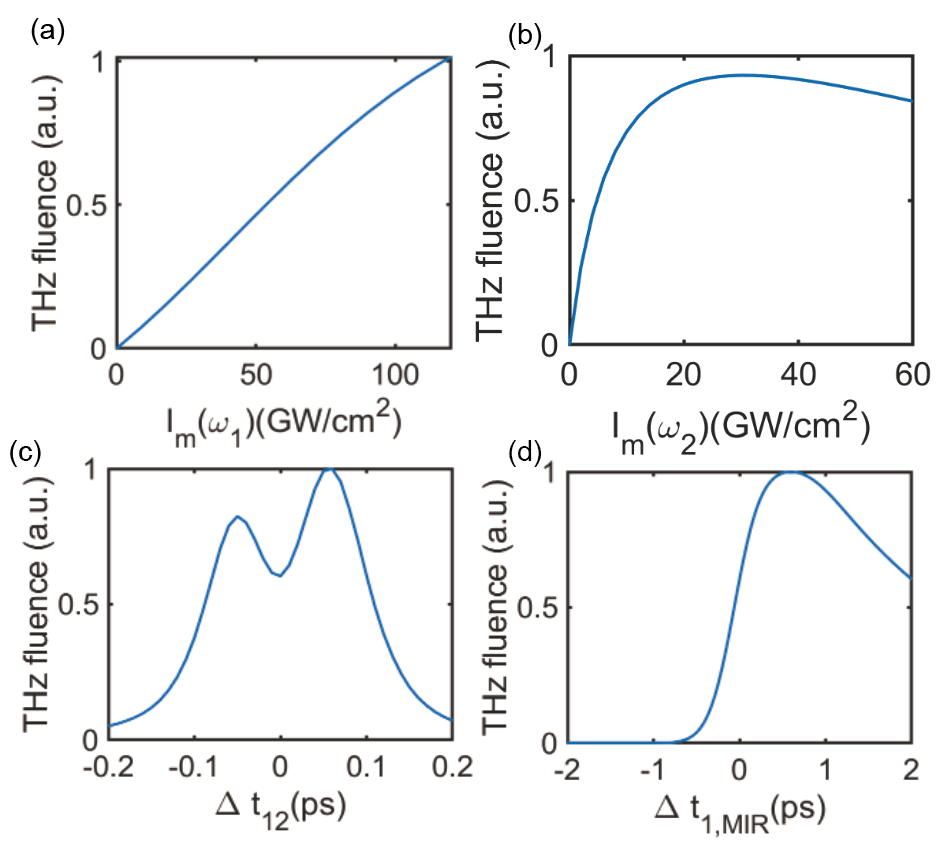


Fig. S THz fluence at the beam center versus (a) (b) (c) (d) . The default parameters in these figures are: , , .

1. **THz output energy versus the crystal length with different *Im*(*ω*1)**


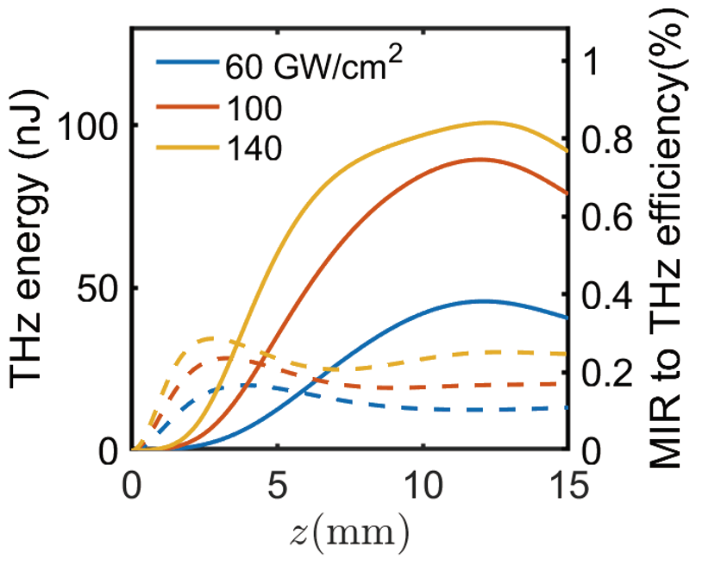


Fig. S The theoretical THz pulse energy versus the crystal length with different *ω*1 peak intensity, while *ω*2 pulse peak intensities *Im*(*ω*2)are fixed at 1 GW cm-2 (1 J per pulse, solid curves) and 80 GW cm-2(80 J per pulse, dashed curves). The laser-induced damage threshold of *ω*1 in diamond is about 140 GW cm-2. We can see that for any input *Im*(*ω*2), the THz output is always positively related to the *Im*(*ω*1) below the damage threshold although the saturation effect over *Im*(*ω*1) can also be found when crystal length is over 10 mm with low *Im*(*ω*2). Therefore, we can theoretically conclude that the optimal *Im*(*ω*1) for the highest THz output should be always close to its damage threshold.

1. **Optimal input conditions for different crystal length**
2. and

For short crystal lengths where the pump depletion and the group velocity mismatch of two pulses in diamond can be neglected, the optimal will move back to 0 fs, and optimal is limited by the damage threshold of , as shown in Fig. S9a where *L* = 1 mm and *ω*1, peak intensity For longer crystal, the THz output versus and , using the experimental beam size, of *L* = 2 mm, 5 mm and 10 mm are shown in, Fig. 4d, Fig. S9b and Fig. S9c. For longer crystals, the depletion becomes more severe so that for the same , the splitting of the Resonant peak along become larger. But the optimal case is similar, as labeled by the black circle in these figures. Decreasing the is the most effective way to increase the THz output for increasing crystal length while the optimal for each length is all around +50 fs.


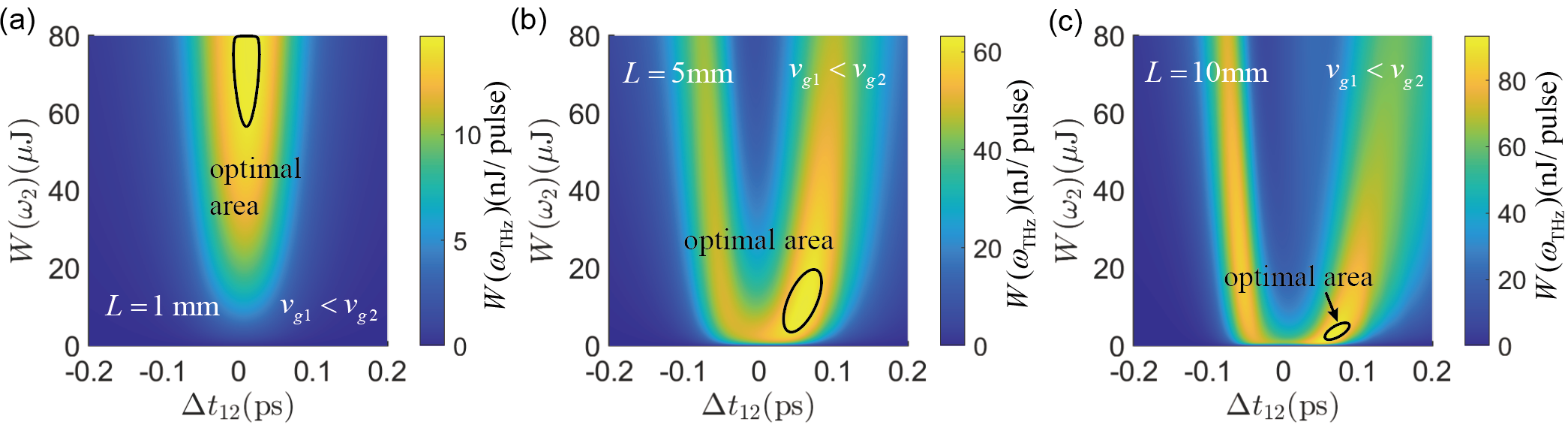


Fig. S (a-c) The whole beam THz output energy versus and with *L* = 1 mm, *L* = 5 mm, 10 mm. The area circled in the black curve corresponds to the area where the THz output is less than 5% from its optimal value.

1. and

Similar to section (a), the global optimal for different crystal lengths is all around 0.6 ps, as shown in Fig. S10a- Fig. S10c. For longer crystal (*L* = 5 mm and 10 mm) and strong , the THz energy oscillation versus can be found, suggesting the strong depletion induced phase fluctuation between 0 to of the phonon phase versus *t*1.


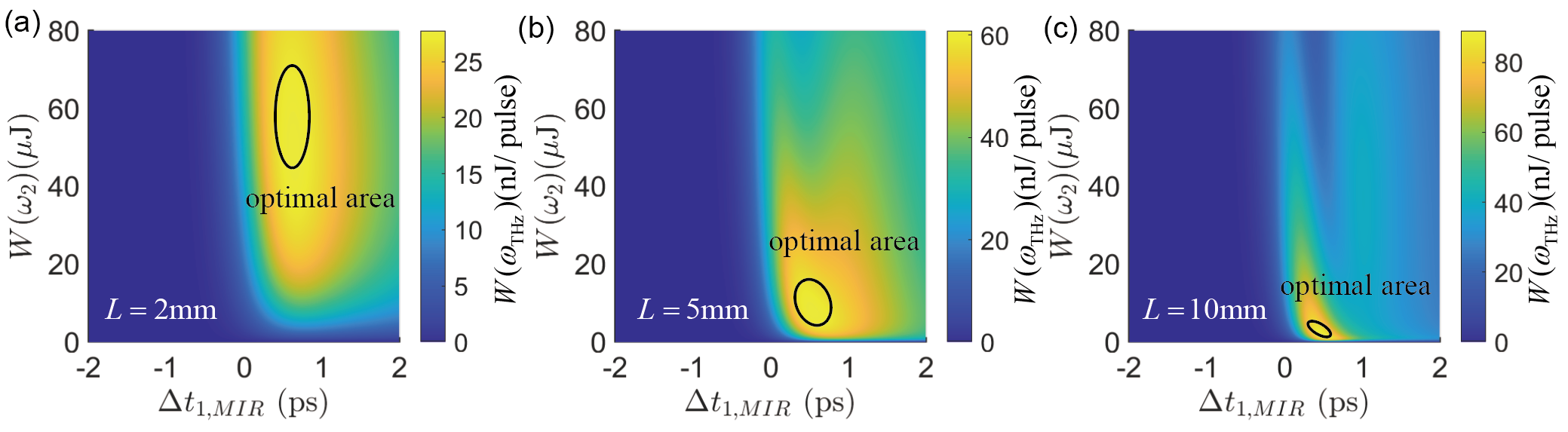


Fig. S. THz output energy versus the and with (a) *L* = 2 mm (b) *L* = 5 mm (c) *L* = 10 mm. The THz output in the optimal area circled in black deviates less than 5% from the optimal value.

1. **A quantitative model about the optimization of the THz output**

To better understand the optimization of the THz output, we should describe the phonon amplitude distribution versus the pump intensity in a more quantitative way. In section (a), we will give the phonon-photon conservation rules and get the up-limit of total phonon number density, from which we can understand why the optimal from different crystal length is all around +0.6 ps. In section (b), we combined the phonon number up-limit with the phenomenon in Fig. 4a- Fig. 4b to estimate the averaged phonon amplitude for given pump intensities, which gives a more quantitative description of Fig. 5a- Fig. 5b.

1. Energy conservation rules and the up limit of the phonon number

The photon density can be expressed by: , and phonon density is . From Eq(7a-b), we can obtain the photon number conservation rules:

Notably, this GVM induced photon number variation can be neglected especially when input pulses are picosecond pulses. The phonon-photon conservation rules can be written by:

Where is the energy loss due to the damping of the Raman phonon.

Integrating Eq. S7 along *z* and *t*, we can get the up-limit of the total phonon number distributed over z at *t*1:

Here, corresponds to the total effective photon number before time *t*1considering the damping loss. Eq. S9 means that the phonon excitation at *t*1 stops when photon before time *t*1 is fully depleted, which is consistent with the physical discussed in Fig. 3. In the experiment, peaks at but when , the available *ω*1 photon for SRS will decrease because of the phonon damping. As a result, we should always fix at 0.6 ps for larger phonon number.

1. Estimation of phonon amplitude distribution *Q*(*z,*)

For simplicity, we approximate the peak function of as a rectangular distributed function: Here, is the averaged phonon amplitude in the range of [0, *Ld*] and *Ld* is effective peak length. Stronger *Im*(*ω*2) will decrease the phonon distribution length, as shown in Fig. 4b in the main text. As a result, the phonon number constrain given in Eq. (S9) becomes: so that the averaged phonon amplitude . We can also conclude that the peak value of is inverse proportion to the square root of peak width.

The THz output can then be estimated to be proportional to , which means we should make the phonon amplitude spread in the whole crystal to maximize the THz output. Longer diamond requires a smaller , as shown in Fig. 5b. The optimal THz output versus is proportional to , positive related to the crystal length, leading to Fig. 5a.

1. **Comparison of different THz pulse generation schemes**

**Table S1 | Summary of THz generation schemes suitable for small laboratory**

| Scheme | Reference | Input parameters | | Output characteristics | | | |
| --- | --- | --- | --- | --- | --- | --- | --- |
|  |  | Wave-length (μm) | Pulse energy (μJ) | Peak freq. (THz) | Bandwidth (THz) | Pulse energy (μJ) | Conversion (‰) |
| OR in LiNbO3 | Ref. 4 | 0.8 | 6.5×103 | 0.35 | 0.5 | 6.4 | 1 |
| Gas plasma | Ref. 5 | 0.8 | 560 | 6 | 12 | ~0.06 **a** | ~0.1 **a** |
| DFG in GaSe | Ref. 6 | 1.1 | ~300 | 30 | 10 | 1.7 | ~6 |
|  |  | ~1.2 | ~300 |  |  |  |  |
| DFG in GaSe | Ref. 7 | 1.03 | ~3 | 13 | 6 | 1.5×10-3, **b** | 0.5**b** |
|  |  | 1.07 | 4.7 |  |  |  |  |
| Collinear CSRS in diamond | this work | 1.45 | 64 | 5 | 7.5**c** | 9.4×10-3, **d** | 0.67 |
|  |  | 1.80 | 55 | 10 |  | 3.5×10-2, **d** | 2.5 |
|  |  | ~6 | 14 | 12 |  | 5×10-3, **d** | 3.6 |

**a**Estimated with 10-4 yield.

**b**Scaled from THz generation at higher frequency in Ref. 13. The pulse repetition rate was 190 kHz.

**c**The bandwidth is defined by full width of half height of the THz energy spectrum.

**d**THz pulse energy in diamond without transmission loss cross diamond/air interface.

**Reference**

1 Le J, Su Y, Tian C, Kung AH, Shen YR. A novel scheme for ultrashort terahertz pulse generation over a gapless wide spectral range: Raman-resonance-enhanced four-wave mixing. *Light Sci Appl* 2023; **12**: 34.

2 Cardona M. Semiconductors under Uniaxial Strain. *Phys. Stat. Sol. (b)* 1996; **198**: 5.

3 Hounsome LS, Jones R, Shaw MJ, Briddon PR. Photoelastic constants in diamond and silicon. *Phys Stat Sol (a)* 2006; **203**: 3088–3093.

4 Wu, X. J. *et al*. Terahertz generation in lithium niobate driven by Ti: sapphire laser pulses and its limitations. *Optics Letters*2014;**39**: 5403-5406.

5 Ho, I. C., Guo, X. Y. & Zhang, X. C. Design and performance of reflective terahertz air-biased-coherent-detection for time-domain spectroscopy. *Optics Express*2010; **18:** 2872-2883.

6 Sell, A., Leitenstorfer, A. & Huber, R. Phase-locked generation and field-resolved detection of widely tunable terahertz pulses with amplitudes exceeding 100 MV/cm. *Optics Letters* 2008;**33:** 2767-2769.

7 Knorr, M. *et al*. Phase-locked multi-terahertz electric fields exceeding 13 MV/cm at a 190 kHz repetition rate. *Optics Letters* 2017;**42**: 4367-4370.
